# GrapevineXL reliably predicts multi-annual dynamics of vine water status, berry growth, and sugar accumulation in vineyards

**DOI:** 10.1093/hr/uhad071

**Published:** 2023-04-13

**Authors:** Weiwei Yang, Junqi Zhu, Cornelis van Leeuwen, Zhanwu Dai, Gregory A Gambetta

**Affiliations:** Beijing Key Laboratory of Grape Science and Enology and Key Laboratory of Plant Resources, Institute of Botany, the Chinese Academy of Sciences, Beijing, 100093, China; EGFV, Univ. Bordeaux, Bordeaux Sciences Agro, INRAE, ISVV, Villenave d'Ornon, 33882, France; The Key Laboratory of Special Fruits and Vegetables Cultivation Physiology and Germplasm Resources Utilization in Xinjiang Production and Construction Group, College of Agriculture, Shihezi University, Shihezi, 832000, China; The New Zealand Institute for Plant & Food Research Limited, Blenheim 7201, New Zealand; EGFV, Univ. Bordeaux, Bordeaux Sciences Agro, INRAE, ISVV, Villenave d'Ornon, 33882, France; Beijing Key Laboratory of Grape Science and Enology and Key Laboratory of Plant Resources, Institute of Botany, the Chinese Academy of Sciences, Beijing, 100093, China; China National Botanical Garden, Beijing 100093, China; EGFV, Univ. Bordeaux, Bordeaux Sciences Agro, INRAE, ISVV, Villenave d'Ornon, 33882, France

## Abstract

Climate and water availability greatly affect each season’s grape yield and quality. Using models to accurately predict environment impacts on fruit productivity and quality is a huge challenge. We calibrated and validated the functional-structural model, GrapevineXL, with a data set including grapevine seasonal midday stem water potential (Ψ_xylem_), berry dry weight (DW), fresh weight (FW), and sugar concentration per volume ([Sugar]) for a wine grape cultivar (*Vitis vinifera* cv. Cabernet Franc) in field conditions over 13 years in Bordeaux, France. Our results showed that the model could make a fair prediction of seasonal Ψ_xylem_ and good-to-excellent predictions of berry DW, FW, [Sugar] and leaf gas exchange responses to predawn and midday leaf water potentials under diverse environmental conditions with 14 key parameters. By running virtual experiments to mimic climate change, an advanced veraison (i.e. the onset of ripening) of 14 and 28 days led to significant decreases of berry FW by 2.70% and 3.22%, clear increases of berry [Sugar] by 2.90% and 4.29%, and shortened ripening duration in 8 out of 13 simulated years, respectively. Moreover, the impact of the advanced veraison varied with seasonal patterns of climate and soil water availability. Overall, the results showed that the GrapevineXL model can predict plant water use and berry growth in field conditions and could serve as a valuable tool for designing sustainable vineyard management strategies to cope with climate change.

## Introduction

The sustainability of crop productivity and quality is threatened by climate change and increasingly frequent extreme events [1]. Temperature is increasing globally, and drought events are occurring more frequently and lasting longer in most regions of the world [1]. When available soil water decreases, plants reduce water use by closing their stomata, limiting plant photosynthesis and plant growth. When water deficits become very severe, plants are subjected to losses of hydraulic conductivity, which increase mortality risks [2,3]. Because productivity losses and mortality risks are related to plant water status [3], it is crucial to precisely predict plant water status in the field [4] in order to accurately predict the associated losses and risks under climate change.

Plant water potential (Ψ) is the most accurate measure of plant water status and is strongly coupled to stomatal regulation and photosynthesis. Stomatal conductance (*g*_s_) and leaf photosynthesis (*P*_n_) response curves to plant water potential are often used to quantify thresholds for a species’ behavior under drought [2, 5–8]. These response curves are used so ubiquitously because they represent the plant’s integration of many complex traits and environmental influences. For example, vascular anatomy and organ topology [7, 9], canopy microclimate (*i.e.* temperature, vapor pressure deficit, radiation, etc. at the canopy level) [10, 11], and maintenance of hydraulic integrity [12, 13], all interact in the resulting Ψ × *g*_s_ and Ψ × *P*_n_ relationships. Therefore, modeling approaches must integrate these complex relationships between the soil, plant, and environment to make accurate predictions.

Various attempts have been made to establish models to predict Ψ, such as non-linear fitting [14], remote sensing [15], and machine learning [16]. Although environment or cultivar characteristics were considered in such models, they are empirical and site-specific. Process-based hydraulic model frameworks described the interplay among leaf- or xylem- and soil water potentials, *g*_s_, and transpiration [17]. These models often presented solely theoretical analysis, or simulations were validated in controlled environments and only over a short period. This is mainly because measurements of the leaf (Ψ_leaf_) and xylem (Ψ_xylem_) water potentials are destructive and laborious [18] and there is a clear lack of long-term databases of plant water potentials in field conditions. In addition, plant hydraulic models often do not consider plant productivity and product quality.

The development of functional-structural plant models (FSPM) offered new opportunities for coupling canopy architecture and multi-scale physiological processes [19]. In grapevine, FSPM models have been used to couple three-dimensional grapevine architecture [20] to leaf-scale photosynthesis and stomatal models, with consideration of variability in canopy, microclimate, and crucial leaf biochemical (e.g. leaf nitrogen content per area, *N_a_*) and physiological (leaf gas exchange) traits [10, 11]. Later, an updated model was used to predict canopy gas exchange more precisely through coupling hydraulic and leaf energy balance models [21]. However, the fruit component, or accounting for carbon allocation among organs, was absent from previous FSPM models. More recently, a FSPM named GrapevineXL [22–24] coupled the broad and multi-scale bio-physiological modules mentioned above and a berry growth module [25]. This model was validated in predicting berry development (e.g. growth and sugar accumulation) under different crop loads in the greenhouse and potted field-grown vines [22–24]. The ability of this model to predict plant water status and berry development under field conditions remained untested.

Grapes are one of the world’s most widely grown and economically important fruit crops. Grape berry fresh weight (FW) and sugar concentration per volume (called [Sugar] hereafter) are important fruit quality traits. Berry FW is the vital component for grape yield and juice volume. [Sugar] determines the alcohol level in the final wine after fermentation and is used as a proxy for other quality-related compounds [26]. Winegrape production is particular in that mild-to-moderate water deficit in vineyards is often considered beneficial because it can suppress overly vigorous vegetative growth and increases berry quality [27, 28]. For example, berry [Sugar] can be increased through repartitioning assimilated carbohydrate to berries, reduced water from soil and water loss from berry skin through berry transpiration; berry anthocyanin concentration and content can be improved by up-regulated expression of genes in the anthocyanin biosynthetic pathway [26–28], etc. Nevertheless, with climate change, increasing temperatures are advancing phenology, increasing [Sugar], but decreasing acidity, anthocyanins, and aroma precursors at harvest, which have the potential to decrease wine quality, producing wines that are unbalanced for alcohols, and lacking acidity, freshness, color, and aroma expression [29–31]. In addition, severe and/or prolonged water deficits can negatively affect grapevine canopy development, berry microclimate, berry size, yield, berry composition, and can increase mortality risk [2, 3, 32]. Thus, predicting seasonal plant water potential is essential to predict berry growth and quality, to evaluate the effect of water availability under various climate scenarios, and to aid growers in developing appropriate mitigation and adaptation strategies to ensure grape yield and quality.

In the current study, we calibrated and validated the GrapevineXL model for a wine grape cultivar (*Vitis vinifera* cv. Cabernet Franc) in field conditions over 13 years in Bordeaux, France to reach the following objectives: (i) to use this model to predict seasonal dynamics of Ψ, *g*_s_, and *P*_n_ under field conditions; (ii) to predict seasonal dynamics of berry dry weight (DW), FW and [Sugar] under field conditions; and (iii) to use the model to predict the impacts of shifting phenology due to climate change on berry growth and sugar accumulation.

## Results

### Predicting plant water status in the field

**Figure 1:**
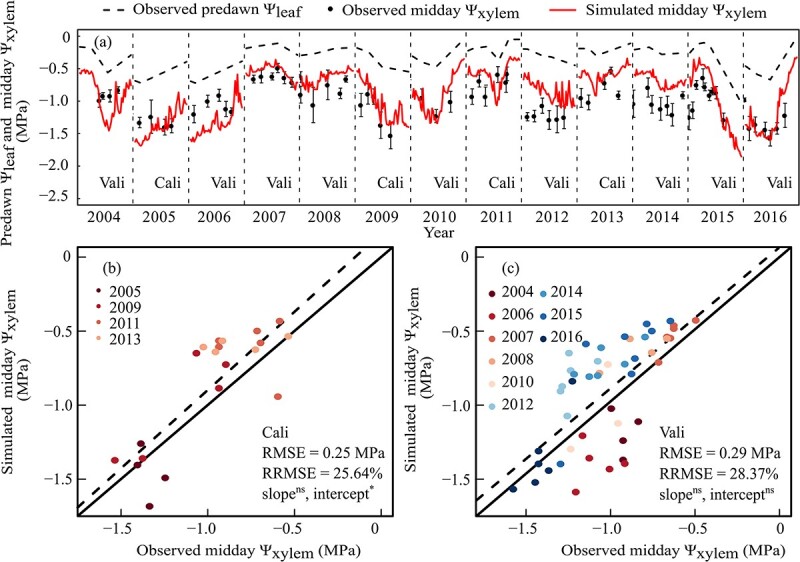
Observed mean midday xylem water potential (Ψ_xylem_) and simulated midday Ψ_xylem_ of grapevine in field from 2004 to 2016. Cali and Vali represent the data belonging to calibration (2005, 2009, 2011, and 2013) and validation (the remaining 9 years) data sets, respectively. The upper panel showed comparisons between observed and simulated midday Ψ_xylem_ over time, with black points for observed midday Ψ_xylem_ (the mean of eight measurements at about 3 p.m.), black dashed lines for observed predawn leaf water potential (Ψ_leaf_), and red solid lines for simulated midday Ψ_xylem_ extracted from the hourly simulated Ψ_xylem_ at 3:00 p.m. (noon) of each day in each vintage. The bottom panels are direct comparisons between observed and simulated midday Ψ_xylem_ for calibration (**b**) and validation (**c**) data sets, respectively. Solid lines in the bottom panels are the 1:1 lines between observed and simulated values and dashed lines are the linear regression lines. ^*^ and ns represent significant or no significant difference in slope and intercept detected between regression line of observed and simulated values and 1:1 line at *P* < 0.05, using ANCOVA.

The Ψ_xylem_ is a sensitive and reliable indicator of plant water status [33], and its fluctuations result from climate factors, soil water availability, and plant hydraulic properties and responses [14]. The seasonal trends of midday Ψ_xylem_ (Fig. 1A) were fairly reproduced by the model after re-parameterization for the *Rsp* and Ψ_50%-leaf_ from 2004 to 2016. For the calibration data set, the RMSE and RRMSE were 0.25 MPa and 25.6%, and the corresponding value was 0.29 MPa and 28.4% for the validation data set. The ANCOVA test showed that there were no significant differences in both the regression slope and intercept from 1:1 line for the validation data set and in regression slope from 1:1 line for the calibration data set. The model performed particularly well in 2007, 2010, 2015, and 2016, while overestimated in 2008, 2014, and 2012 or underestimated in 2004 and 2006 (Fig. 1B and C).

### Model performance of leaf-scale gas exchange in field with a wide spectrum of soil water availability

**Figure 2:**
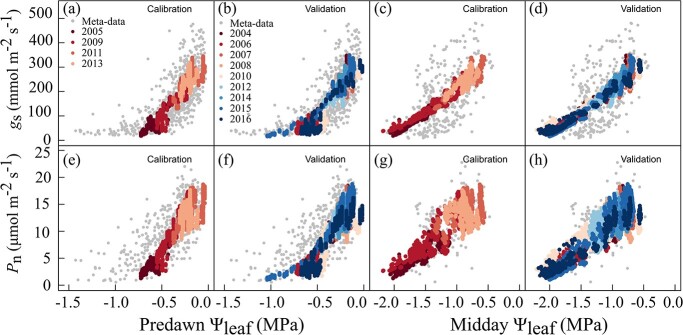
Observed and simulated responses of stomatal conductance (*g*_s_), and net photosynthesis rate (*P*_n_) to predawn leaf water potential (Ψ_leaf_) and midday Ψ_leaf_ using GrapevineXL model, respectively, in field from 2004 to 2016. Calibration and Validation represent the data belonging to calibration (2005, 2009, 2011, and 2013) and validation (the remaining 9 years) data sets, respectively. Grey points are data from published literatures (Table S1, see online supplementary material), representing observed values. Colorful points are simulated values for calibration and validation data sets, respectively.

In order to calibrate and validate the model performance of leaf-scale gas exchange simulation in field conditions for a wide spectrum of soil water availability, a meta-analysis of 18 field studies published between 1996 and 2019 was used (Fig. 2; Table S1, see online supplementary material). In the published studies, the leaf gas exchange and corresponding midday Ψ_leaf_ were usually measured on sun-exposed and fully expanded leaves around midday or time was not specified but with saturated light. To make reliable comparisons, simulated leaf gas exchanges from GrapevineXL were extracted from the top 3 leaves on the shoot around midday between 12 p.m. and 4 p.m., because these leaves have similar light conditions as those in the published studies. Model parameters of *leafN_content*, *slope_V_cmax_,* and Ψ_50%-leaf_ that regulate leaf *P*_n_ and *g*_s_ were first refined by model calibration. For the calibration data set, GrapevineXL precisely reproduced the responses of *g*_s_ and *P*_n_ to predawn Ψ_leaf_, with simulated points nicely overlapping with experimental observations across a wide range of predawn Ψ_leaf_ (−0.03 to −0.72 MPa) (Fig. 2A and E). For the validation data set, GrapevineXL also accurately predicted the responses of *g*_s_ and *P*_n_ to predawn Ψ_leaf_, within an even wider predawn Ψ_leaf_ range (−0.01 to −1.03 MPa) (Fig. 2B and F). Similarly, the model generated a comparable range of midday Ψ_leaf_ to those observed in the meta-analysis and accurately predicted the responses of *g*_s_ and *P*_n_ to midday Ψ_leaf_ which distributed within the area of the observed meta-analysis for both calibration and validate data sets (Fig. 2C, D, G, and H). Moreover, the model correctly reproduced the decreases of *P*_n_ and *g*_s_ gradients when water limitation intensified (Fig. 2). The general adequacy of responses of *g*_s_ or *P*_n_ to predawn Ψ_leaf_ or midday Ψ_leaf_ between predicted and observed meta-analysis values across both diverse climate and soil water conditions indicated a robust predictive capability of GrapevineXL.

### Predicting changes in berry fresh weight and sugar concentration

**Figure 3:**
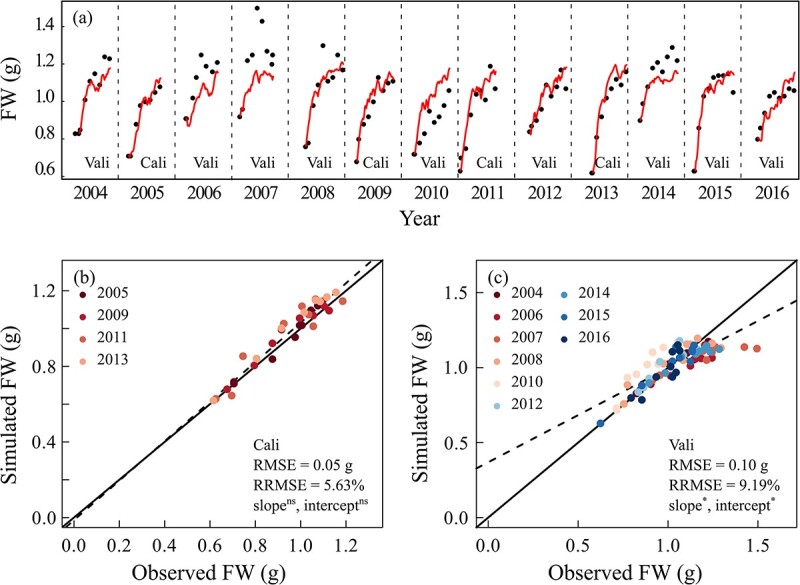
Observed and simulated berry fresh weight (FW) using GrapevineXL model in field from 2004 to 2016. Cali and Vali represent the data belonging to calibration (2005, 2009, 2011, and 2013) and validation (the remaining 9 years) data sets, respectively. The upper panel showed comparisons between observed and simulated berry FW over time, and the bottom panels were direct comparisons for calibration and validation years, respectively. Each point represented the average value of all berries from the simulated vine. Black points in the upper panels are observed berry FW and red solid lines are simulated values. Solid lines in the bottom panels are the 1:1 lines between observed and simulated values and dashed lines are the linear regression lines. ^*^ and ns represent significant or no significant difference in slope and intercept detected between regression line of observed and simulated values and 1:1 line at *P* < 0.05, using ANCOVA.

**Figure 4:**
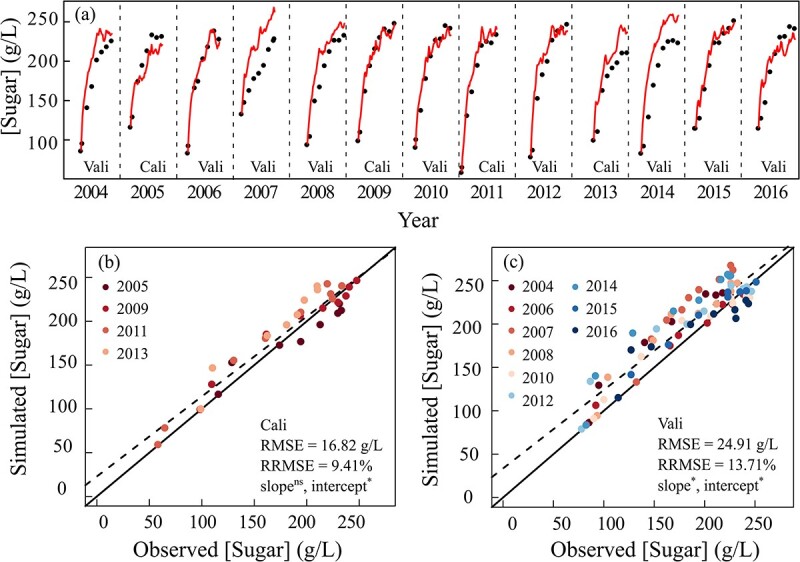
Observed and simulated berry sugar concentration ([Sugar]) using GrapevineXL model in field from 2004 to 2016. Cali and Vali represent the data belonging to calibration (2005, 2009, 2011, and 2013) and validation (the remaining 9 years) data sets, respectively. The upper panel shows comparisons between observed and simulated berry [Sugar] over time, and the bottom panels are direct comparisons for calibration and validation years, respectively. Each point represents the average value of all berries from the simulated vine. Black points in the upper panels are observed berry [Sugar] and solid lines are simulated values. Solid lines in the bottom panels are the 1:1 lines between observed and simulated values and dashed lines are the linear regression lines. ^*^ and ns represent significant or no significant difference in slope and intercept detected between regression line of observed and simulated values and 1:1 line at *P* < 0.05, using ANCOVA.

Following predictions of leaf-scale gas exchange and plant water status (Figs 1 and 2), the model accurately reproduced the seasonal dynamic patterns of berry DW, FW, and [Sugar] compared to observed values for both calibration and validation data sets (Figs 3 and 4; Fig. S1, see online supplementary material). For the calibration data set, there was a slight underestimation of DW in 2005 and 2009 and an overestimation of [Sugar] in 2013. The ANCOVA test showed that there were no significant differences in both the regression slope and intercept of FW and in the regression line slope of [Sugar] from 1:1 line, but the intercept was significantly different for the regression line of [Sugar] from 1:1 line, for the calibration data set. The RMSEs were 0.02 g, 0.05 g, and 16.82 g/L and RRMSE were 7.31%, 5.63%, and 9.41% for DW, FW, and [Sugar], respectively. For the validation data set, the [Sugar]s were overestimated in 3 years (2004, 2007, and 2014), and the corresponding DW and FW were both underestimated, at the later stage of growth. Significant differences in both the regression slopes and intercepts of FW and [Sugar] from 1:1 line were detected according to ANCOVA test for the validation data set. The RMSEs were 0.02 g, 0.01 g, and 24.91 g/L and RRMSEs were 8.89%, 9.19%, and 13.71% for DW, FW, and [Sugar]. Based on the value of RRMSEs of validation data sets, the model showed excellent performance in predicting berry growth and good performance in prediction berry sugar accumulation. Moreover, inter-annual variations in berry FW, [Sugar], and DW at maturity were also precisely predicted, without significant differences between observed and simulated values at maturity (Fig. S2, see online supplementary material).

### Simulating the impacts of phenology shifts on berry size and sugar concentration

Virtual experiments were conducted to explore the effects of earlier veraison due to climate warming [34] on berry growth, sugar accumulation, and ripening phase between veraison and harvest. Two early veraison scenarios were created by moving forward the veraison dates by 14 and 28 days in comparison with the observed veraison dates from 2004 to 2016. When the veraison was advanced by 14 or 28 days, one would expect to observe a 14-day or 28-day advancement in maturity date as well, if the ripening duration was not modified. However, temperature may modify the duration from veraison to maturity [35]; therefore, the advancements of maturity under an early veraison scenario might be larger or smaller than 14 or 28 days. Because the actual advancements of maturity could not be known *a priori*, we considered the same duration from veraison to harvest for running simulations in the two virtual scenarios. When the date of veraison moved forward, ripening took place under warmer conditions, as the number of days that had higher temperature and radiation was higher than the default scenario (Fig. S3, see online supplementary material), leading to higher GDDs (Fig. S4, see online supplementary material), in all years except 2011. Compared to default scenarios, except in 2011, the GDD increased by 2.4 to 111.4°C days for the eVer_14 scenario, and by 32.2 to 139.6°C days for the eVer_28 scenario (Fig. S4, see online supplementary material). For 2011, the GDD was decreased by 30.8°C days for the eVer_14 scenario and 42.3°C days for the eVer_28 scenario, respectively. On average, increases of 43.4 and 75.4°C days were detected for eVer_14 and eVer_28 scenarios, respectively.

**Figure 5:**
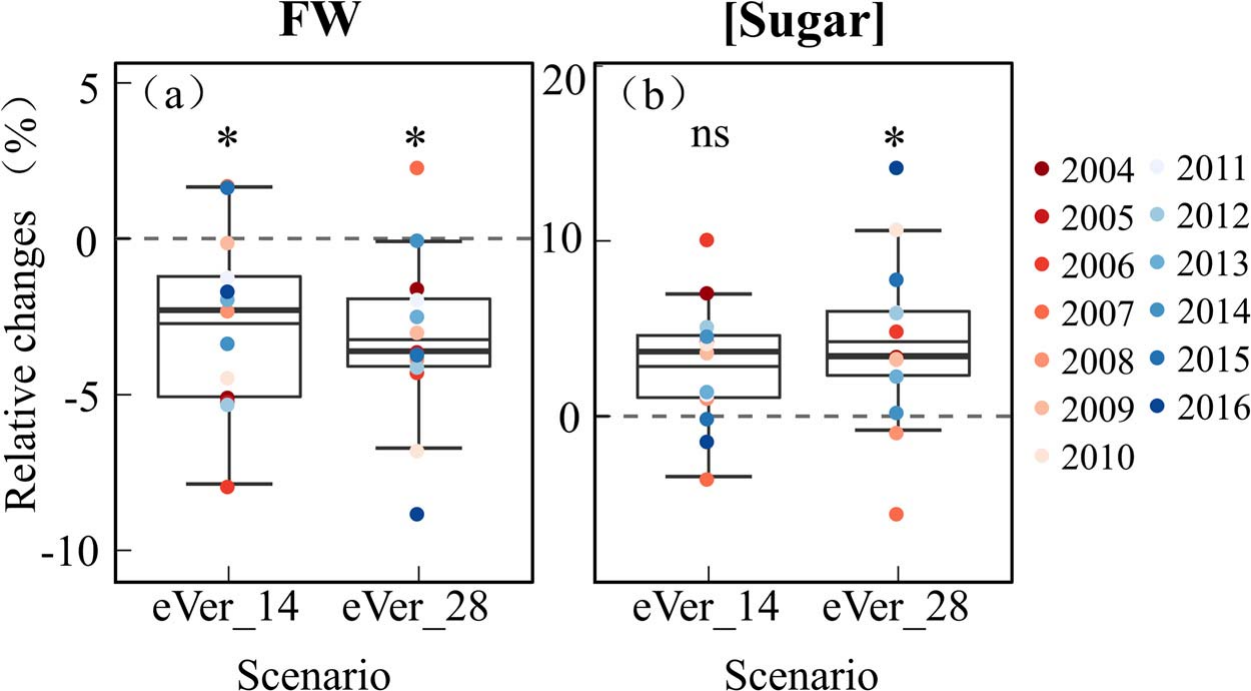
Relative changes in simulated berry fresh weight (FW) and sugar concentration ([Sugar]) over 13 years from 2004 to 2016, using GrapevineXL model in field conditions, under earlier veraison scenarios. The simulated berry FW and [Sugar] under default veraison were used as the baseline in each year to estimate the relative changes of values simulated under 14- and 28-days earlier veraison scenarios, respectively. The boxes and whiskers represent the variation in relative changes among years. The thick and thin dashes in boxes are median and mean of all values, and colorful dots are simulated results. ^*^ and ns represent significant difference or no significant differences detected between simulated values under default and earlier veraison scenarios at *P* < 0.05, using one-way ANOVA.

The berry DW, FW, and [Sugar] under earlier veraison scenarios were compared to the default scenario (Fig. 5). Their relative changes varied among years and the two scenarios. FW decreased in 11 out of 13 years under eVer_14 scenario and in 12 out of 13 years under eVer_28 scenario, while it was increased in 2007 and 2015 under eVer_14 scenario and in 2007 under eVer_28 scenario. Inversely, [Sugar] increased in 10 out of 13 years under eVer_14 scenario and in 11 out of 13 years under eVer_28 scenario, while it was decreased in 2007, 2015, and 2016 under eVer_14 and in 2007 and 2008 under eVer_28 scenario. Over the 13 years, FW was significantly reduced by 2.7% for eVer_14 and by 3.22% for eVer_28 scenario compared to the default. [Sugar] increased by 2.90% for eVer_14 and significantly increased by 4.29% for eVer_28 scenario compared to the default. Over the 13 years, the highest increase of [Sugar] was 10.11% in 2006 under eVer_14 and 14.24% in 2016 under eVer_28 scenarios. The FWs in the two years also showed the highest decreases over the 13 years, which were − 7.93% in 2006 under eVer_14 and − 8.82% in 2016 under eVer_28 scenarios. There were no significant differences for DW between the earlier and default scenario (Fig. S5, see online supplementary material).

**Figure 6:**
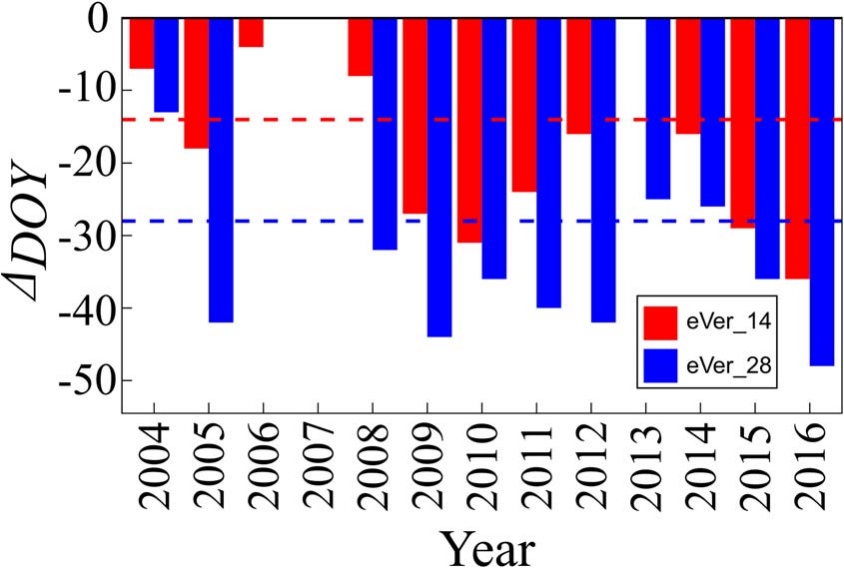
Relative changes of simulated DOY for berry ripe under earlier veraison scenarios compared to default veraison scenario over 13 years from 2004 to 2016, using GrapevineXL model in field. The simulated berry maturity DOY under earlier veraison scenarios were determined as the day when berry [Sugar] reached the largest [Sugar] under default veraison scenario. Red and blue bars present the changes of DOY for berry ripe under 14- and 28-days earlier veraison scenarios, respectively. Red and blue lines present 14- or 28-day advancements in maturity, respectively.

Grape maturity is often determined by growers based on various criteria with the consideration of target wine styles [26]. Among those criteria, [Sugar] is one of the most important indexes and the date that berries reach a target [Sugar] has been used as a reliable proxy for evaluating historical changes in grape maturity dates [36]. Similarly, we calculated berry maturity date (DOY) under advanced veraison scenarios as the time when the simulated berry reaches the highest sugar concentration of default scenario in each year (Fig. 6). Then the relative changes of maturity date (*Δ*_GDD_) under earlier veraison scenarios were compared to the default scenario (Fig. 6). Three possible response patterns of ripening duration and maturity date can theoretically occur: (i) Since we advanced veraison date by 14 or 28 days, one would expect equal advances in maturity date if the ripening duration was not affected. However, the advancement in veraison will most likely modify growing temperatures during ripening, hence influencing ripening rate and consequently altering ripening duration. Therefore, (ii) if the ripening period is shortened, then the maturity date will be advanced more than the days advanced in veraison (namely 14 or 28 days); in contrast if the ripening period is prolonged, (iii) the maturity will be advanced less than 14 or 28 days. Our simulation results showed that the ripening period in 8 years (2005, 2009 to 2012, and 2014 to 2016) out of 13 was shorter than default under the eVer_14, while in 3 years (2004, 2006, and 2008) out of 13 was longer than default. Similar results were obtained for the eVer_28, with 8 years (2005, 2008 to 2012, 2015 and 2016) having shorter ripening period and, and 3 years (2004, 2013, and 2014) having a longer ripening period. It is also noteworthy that the [Sugar] in 2006 under eVer_28, 2013 under eVer_14, and 2007 under both virtual scenarios never reached the target [Sugar] in the default scenario, and therefore no maturity dates were determined for those instances.

## Discussion

In this study, the functional-structural plant model, GrapevineXL, was calibrated and validated against observed data of Cabernet Franc grapevines in Saint-Émilion (Bordeaux, France) over 13 consecutive years under varied soil water and climate conditions. The results demonstrated that the model could accurately predict the dynamics of grapevine water status and berry DW, FW, and [Sugar]. The simulated leaf *g*_s_ and plant Ψ_xylem_ declined mainly following the decline of predawn Ψ_leaf_ but was also influenced by climate conditions. Exploring advanced phenology due to climate change suggests that in general this advancement will decrease berry size and increase sugar concentration, consistent with the observed trends to date. Overall, the results showed that the multiscale GrapevineXL model can predict plant water use and berry growth, and provides a useful tool predicting grapevine growth and productivity under future climate scenarios.

### Predicting plant water status with soil water availability and climate

Plant water status is a major driver of key physiology processes that influence plant production and product quality [3], and could be reliably evaluated with Ψ_leaf_ and Ψ_xylem_. Because Ψ_leaf_ is more dependent on leaf microclimates within canopies than Ψ_xylem_ [18], there is an increasing trend to use Ψ_xylem_ as a plant water status index for irrigation management and evaluating plant performance [14]. Traditionally, Ψ_xylem_ is measured with destructive sampling and is laborious. However, precise and reliable *in situ* measurements of Ψ_xylem_ are still very challenging, while measuring meteorological factors (including light, temperature, and relative humidity) and soil water content are relatively easy. Therefore, it is extremely valuable to develop predictive approaches with just climate and soil water as inputs to provide accurate prediction of plant water status, particularly the Ψ_xylem_.

After re-parameterization and 3D canopy reconfiguration, the GrapevineXL has fair performance in predicting the seasonal dynamics of plant water status (Ψ_xylem_) in the field over 13 vintages. Although Ψ_xylem_ was overestimated (2008, 2012, and 2014) or underestimated (2004 and 2006) across simulated periods in several vintages, it was predicted quite accurately in many vintages (2005, 2007, 2009, 2010, 2015, and 2016). This is mainly attributed to the model’s comprehensive consideration of the whole-plant water fluxes across the soil–plant–air continuum, with special attention to cornerstone pathway hydraulic conductivities, including the soil-to-root hydraulic conductivity (*R*_sp_) and g_s._ The *R*_sp_ is regarded as the primary driver of alternations in plant water status via its influence on *g*_s_ responses under water limitation [37, 38]. In the current study, *R*_sp_ is simulated with two components, including root architecture and soil hydraulic conductivity (Eq. 1). First, the original three-parameter description of root architecture was simplified into one composite parameter, *RSC* (Eq. 1). The use of *RSC* simplifies the calibration burden without loss of prediction precision and compensates for the fact that making accurate non-destructive measurements of root system architecture underground in the field is simply not possible [17]. Second, the variation of soil hydraulic conductivity [*k*(Ψ_soil_)] as a function of soil water availability was calibrated for the specific soil where current experiment was conducted. By doing so, the model captures the variable hydraulic conductance of the whole vine, which is essential for reliable water status simulation as previously shown [39]. Soil type is the main factor influencing soil hydraulic conductivity [40] and the re-parameterization of *k*(Ψ_soil_) is indispensable for the precise prediction of Ψ_xylem_ in vineyard. In the future, this parameter can be tailored to other specific vineyards and soils.

A robust simulation of *g*_s_ under constantly changing environments (e.g. in the field) is also required for a precision prediction of plant water status. Because *g*_s_ is the hub regulator simultaneously controlling *P*_n_ and water fluxes, we re-parameterized three parameters (*leafN_content*, *slope_V_cmax_,* and Ψ_50%-leaf_) related to those two processes with a meta-analysis of field studies. The leaf nitrogen content (*leafN_content)* is known to be largely influenced by light conditions [10], and was adjusted in the present study to represent light differences between the greenhouse and field conditions. The most critical and sensitive parameter for *g*_s_ is the Ψ_50%-leaf,_ which represents the leaf water potential when 50% of the leaf hydraulic conductivity is lost [21, 41]. The Ψ_50%-leaf_ was calibrated in the current study to −1.80 MPa (Table S1 and Fig. S6, see online supplementary material) in order to reproduce the relationship between Ψ_leaf_ vs *g*_s_, Ψ_leaf_ vs *P*_n_, as well as predawn Ψ_leaf_ vs *g*_s_, predawn Ψ_leaf_ vs *P*_n_ in field conditions. This value was more negative than the original value (−1.52 MPa) for greenhouse grapevines [22] and other reports in literature [42, 43]. In the literature, Ψ_50%-leaf_ ranged from −0.86 to −1.61 MPa as a function of cultivar, rootstock, soil type, and planting region or even between *g*_s_ measurement equipment (porometer vs infra-red gas analyzer) [42]. The more negative Ψ_50%-leaf_ value (−1.80 MPa) in the current study avoids a too-steep decrease in *g*_s_ with decreases in Ψ_leaf_, and maintains a relatively low but stable *g*_s_ under severe water stress (e.g. Ψ_leaf_ < −1.52 MPa, Fig. S6, see online supplementary material) similar to those observed in our field meta-analysis. This is in line with recent works that showed that grapevine *g*_s_ becomes increasingly tolerant to more negative water potentials throughout the growing season [44, 45].

Because Ψ_xylem_ could be fairly predicted by GrapevineXL in the field, we further explored the relative contributions of environmental drivers that could influence the fluctuations in Ψ_xylem_. The current study supports previous observations that the decline of Ψ_xylem_ mainly followed the decline of predawn Ψ_leaf_, and 29% of the variability in Ψ_xylem_ could be explained by predawn Ψ_leaf_ (Fig. S7, see online supplementary material). However, the percentage is lower than previous reports that showed about 61% of the variability in Ψ_xylem_ was explained by predawn Ψ_leaf_ for peach [46], 66%–85% for grape [14, 47], and 75% to 81% for pecan [48] where predawn Ψ_leaf_ and midday Ψ_xylem_ were measured in pairs in each day. Thus, the lower percentage in the current study may be as a result of the predawn Ψ_leaf_ not being measured every day and thus a portion of those values were estimated using linear interpolation. Additionally, part of the residual variability in Ψ_xylem_ resulted from climate factors, such as vapor pressure deficit, temperature, solar radiation, etc. [18]. Moreover, the canopy size and structure, and crop load may also affect Ψ_xylem_ via their effects on *g*_s_ [26]. For example, failure to account for the variability in microclimate within canopies led to a 25% overestimation of canopy transpiration in red maple, which would consequently lead to biased results of Ψ_xylem_ [49]. Therefore, the reconfiguration of the 3D grapevine canopy structure likely contributed to the improved prediction precision of Ψ_xylem_. This 3D architecture enables the leaf gas and energy exchange to be accurately simulated at the leaf scale, taking into consideration the variability in microclimate and acclimation of leaf biochemical and physiological key parameters within complex canopies [10].

### Variation in berry weight and sugar

Both berry weight and [Sugar] are complex traits with high plasticity in responses to climate factors and soil water [26, 28, 35, 50]. As a result, they often show high inter-vintage variation [50, 51], which negatively impacts growers in terms of unstable yield and quality. In our data set, berry fresh weight varied from 1.0 to 1.3 g and [Sugar] from 200 to 250 g/L at maturity over the 13 vintages. A framework that is able to predict these inter-annual variations will be valuable for developing and implementing anticipative management strategies for vineyards, such as irrigation, in the short term (i.e. within a season) and for evaluating viticulture sustainability of a given region under climate change in the long term [50, 52].

Following the robust predictions of plant water status and carbon assimilation (*P*_n_), GrapevineXL accurately simulated berry FW and [Sugar] under a range of climatic and soil water conditions with the same set of parameter values across 13 vintages. This was achieved by a minimal re-parameterization of six parameters related to water and carbon fluxes between vines and berries. Those water and carbon influxes and effluxes are modelled using bio-physical and biochemical laws with parameters that are expected to be genotype-dependent but environment-independent [23, 53]. The current results confirmed the stability of model parameters and enable GrapevineXL to serve as a prediction tool for accurately evaluating grape production and sugar accumulation responses under future climate scenarios.

### Advanced veraison changes climatic and soil water pattern conditions, impacting on berry growth and sugar accumulation

Advancement of phenology, such as veraison or harvest, are well-documented effects of climate change in grapevine [34, 54, 55], and result in part from increased temperature and decreased soil water availability [54]. In parallel, grape quality (e.g. [Sugar], [pH], [organic acids]) is also profoundly modified [30, 31]. The advancement in phenology may shift the ripening window and consequently exacerbate the changes in temperature and soil water experienced by the vines [56]. However, accurately quantifying the effects of advanced phenology on berry growth and quality has proven challenging because it results from simultaneously changing both the timing and climate surrounding the ripening process. To disentangle these effects, we designed a series of virtual scenarios of advanced phenology and evaluated the effects of advanced phenology on grape growth and quality, and ripening duration. The scenarios allowed us to mimic the advanced phenology and compare its effects over multiple seasons exhibiting diverse climate and soil water conditions. The simulations in the current study generally agreed with the long-term observed changes to date [30, 31, 34, 56], (i) the climates under earlier veraison were warmer in almost all years (12 out of 13 years, Fig. S5, see online supplementary material); (ii) berry [Sugar]s were increased (11 out of 13 years under eVer_14 and 12 out of 13 years under eVer_28 scenarios, respectively, Fig. 5) and the ripening period from veraison to maturity was shortened (both 10 out of 13 years under both eVer_14 and eVer_28 scenarios, Fig. 6); and (iii) the magnitude of relative changes of berry [Sugar]s and ripening phase varied with climate and soil water patterns.

Moreover, our simulation showed decreases in berry size in most vintages in response to advanced veraison. This is most likely a result of reduced berry water balance rather than decreased source carbon supply. First, the water influxes into berry were reduced because the ripening period took place under more water stressed conditions with advanced veraison [26, 31]. Second, berry water loss via transpiration was increased because the evapotranspiration demand was higher due to higher temperature with advanced veraison [57]. These two processes both reduce water accumulation in berry and thus decrease berry size. On the other hand, the leaf photosynthesis rate, a measure of source carbon supply [7], was not reduced but increased (Fig. S8, see online supplementary material), despite the lower plant water status under advanced veraison. This is because the negative effect of lower plant water status on *P*_n_ might be fully compensated by the higher leaf temperature and leaf absorbed radiation under advanced veraison (Fig. S9, see online supplementary material). Interestingly, the improved *P*_n_ under advanced veraison did not result in increases in berry dry weight, which in fact decreased in most simulations (nine out of 11 years under eVer_14 and seven out of 11 years under eVer_28 scenarios, respectively, Fig. S3, see online supplementary material). This uncoupling between *P*_n_ and berry DW suggests that the carbon partitioning among organs is altered and more carbon was allocated to shoot and roots than to berry and fine roots under water stress in grapevine (Fig. S10, see online supplementary material). This is in agreement with observations in grapevine under drought conditions [58]. These counterintuitive results demonstrate the ability of GrapevineXL to elucidate the complex carbon assimilation and partitioning patterns resulting from interactions among environmental factors and biological processes.

The current modeling approach demonstrated that the effects of shifting phenology on berry growth and quality [28, 35] could differ season to season depending on the specific seasonal weather and soil water patterns [30, 35]. A temperature-only based model cannot fully explain this variability [26, 35] and may not be adequate in differentiating the effects of regional climates and evaluating impacts of climate change.

### Limitations, drawbacks, and transferability of GrapevineXL

This study revealed complex interactions among climate, soil, canopy architecture, and root functions [28, 35, 42]. Missing any one of these interactions because of model simplification may impact model accuracy. Two simplifications had been applied as model assumptions in the current version of GrapevineXL, including constant leaf size and leaf number post-veraison independent of seasonal conditions, and a simplified topology of canopy structure consisting of isolated single shoots with their own roots. Although the canopy size was largely maintained as a result of frequent summer pruning, potential increases of leaf area and number from leaves on the secondary shoots under favorable climate and soil water conditions can occur [59, 60]. Therefore, the assumption of constant canopy size post-veraison may lead to underestimation of canopy transpiration under favorable climate and soil water conditions [61] and then overestimate plant water status, such as the overestimation of Ψ_xylem_ in years (e.g. 2007, 2008, 2011, 2013, and 2014) with less stressed conditions. Although there is vessel segmentation among shoots and water flows in discrete xylem sectors along the trunk axis [62], the assumption of separated shoots ignores the possible exchange of resources between nearby vessels [24] and may increase the uncertainty of predicting plant water status. Detailed leaf growth and canopy structure need to be more precisely incorporated in the future version of the GrapevineXL.

Following the sensitivity analysis in the previous studies [23, 38], the results from the current model demonstrated that re-calibration of key parameters (14 of the total 114 parameters) is adequate and can avoid the need for destructive measurements of roots (for *RSC*)*.* Moreover, through integration of phenology [36], leaf area development [59], and soil water balance [63] models as new modules of GrapevineXL, the required inputs that were obtained by field observations in the current model can be reduced and estimated from meteorological factors. However, the newly introduced parameters from these new modules increase the number of parameters in the current model. In addition, the missing interaction between leaf area and climate and soil can be compensated at the same time.

The current model was calibrated and validated for only one cultivar under rain-fed conditions in a specific region. The results from the model suggested that water and carbon fluxes related parameter were cultivar- and soil- rather than climate-dependent. Using different grape cultivars to adapt to climate change has been proposed as one effective solution [64] and the high genetic diversity within *Vitis vinifera* makes this option promising. However, whether the GrapvineXL can account for this cultivar-dependent diversity needs to be recalibrated with consideration of soil type. By taking advantage of the model, virtual experiments can be efficiently conducted to screen desired traits and provide insights on selection and breeding of potential cultivars to meet the requirements of a region’s changing climate [65].

### Conclusion

This study calibrated and validated the functional-structural grapevine model, GrapevineXL, in field conditions to predict seasonal plant water transport through the soil-root-xylem-leaf continuum (leaf *g*_s_, Ψ_leaf_, and Ψ_xylem_) and berry growth and quality (FW, DW, and [Sugar]) against observed data across diverse climate and soil water conditions. Moreover, the simulations advancing the veraison date indicate the current model provide comparable yet more nuanced results when compared to long-term observations or temperature-based models regarding the impacts of climate change on berry growth and quality. The current study showed this process- and structure-based modeling approach can be effectively applied to current and future climate predictions to aid farmers in developing sustainable adaptation strategies.

## Materials and methods

### Overview of the GrapevineXL model

**Figure 7:**
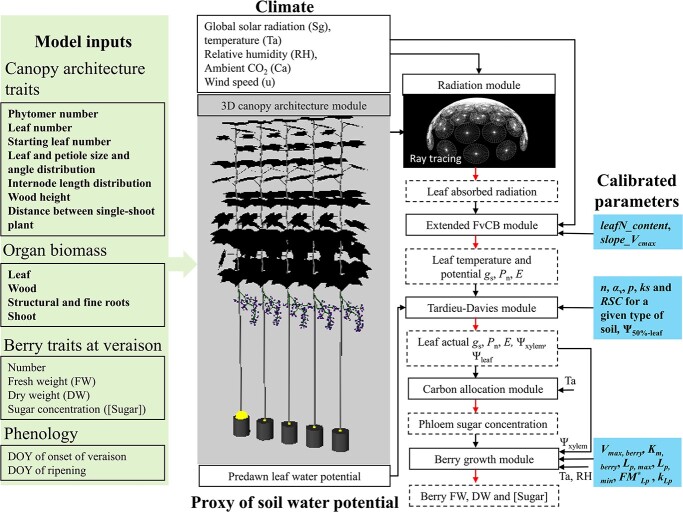
Schematic diagram of the GrapevineXL model. Soil water potential was represented by predawn leaf water potential in current study. Model refinements were highlighted in blue, including adjusting the equation for the soil-to-root conductivity (*RSC*) and parameters recalibration for those related to leaf photosynthesis, soil-root water hydraulics, and berry growth and sugar accumulation, in order to better capture the performance of vines in field. Details of calibrated parameters can be found in the ‘Model parameterization’ section.

GrapevineXL is a whole-plant functional-structural grapevine model designed for simulating post-veraison berry growth that starts from veraison (the onset of ripening) and ends at maturity in response to canopy architecture, leaf-to-fruit ratio, climate, and soil conditions [22,23]. GrapevineXL was developed on the GroIMP platform [66]. There are six main modules in the model (Fig. 7): (i) radiation module which calculates the light captured by each organ using a ray-tracing method [66]; (ii) canopy architecture module which realistically represents the plant 3D architecture; (iii) leaf gas exchange module which calculates the photosynthesis, transpiration, leaf temperature, and stomatal conductance at a given environmental condition using an extended Farquhar, von Caemmerer and Berry (extended-FvCB) method [67]; (iv) water transport module which calculates water transport from soil to root surface to xylem and to leaf with an electrical resistance analogy and with variable hydraulic conductance using the Tardieu–Davies method [68]; (v) carbon allocation module which calculates the phloem sucrose concentration and carbon allocation to each organ on an hourly basis [69, 70]; and (vi) berry growth module which simulates the hourly water and carbon balances of a mean berry based on biophysical laws [23, 71].

In GrapevineXL, the vine architecture is composed of multiple organs that include: 1-year-old stems including internodes, petioles, and leaves, a cordon (more than 2-year-old), a trunk (its weight including structural thick roots), fine roots (less than 2 mm in diameter), and berries (mean individual berry times the number of berries per shoot). All organs are initialized with corresponding biomass and 3-dimensional information (geometry and topology) except the root compartment, which is described as a carbon sink with its corresponding biomass.

The 3D canopy architecture allows a fine simulation of radiation absorbed by organs within a heterogeneous canopy, using a ray-tracing method [66]. By mechanistically coupling the extended-FvCB and Tardieu–Davies models, the model is capable of predicting leaf-scale *P*_n_, *g*_s_, and Ψ_leaf_, Ψ_xylem_ and the dynamic water transport from soil to xylem and to leaves. Then, a sink-driven carbon allocation model is employed to calculate phloem [Sugar] based on the hypothesis that carbon-loading equals carbon unloading among organs at each time step at the whole-plant scale. Finally, phloem [Sugar] and xylem water potential are used by the berry growth module to simulate berry growth and [Sugar].

### Experimental data

A commercial rainfed vineyard located at Saint-Émilion region, France (44.93° N, 0.18° W), was selected as an experimental plot. The studied *Vitis vinifera* L. Cabernet Franc was established in 1998 and grafted on 101-14MGt rootstock at a density of 5950 vines/ha (row spacing at 1.4 m and vine spacing on the row at 1.2 m). Vines were trained to a system with an average of five shoots per vine, ranging from four to eight shoots per vine. The soil in the vineyard was categorized as heavy clay and the rooting depth could reach 1.3 m with a moderate soil water-holding capacity of 168 mm. Vines were simple guyot pruned. All vines were managed according to the standard local practices in the winery, including standard fertilization (only organic), pest and disease protection, and no irrigation was applied. The sampling area was restricted to an area of 100 vines, close to the location where the soil was characterized by means of a soil pit. The rationale to restrict the sampling area was to avoid heterogeneity induced by soil variations (texture, depth, water-holding capacity). Further details of the experimental setting are described in Tramontini *et al.* [72].

Over 13 consecutive vintages (from 2004 to 2016), both predawn Ψ_leaf_ and midday Ψ_xylem_ were measured with a pressure chamber (SAM Précis 2000, F-33170 Gradignan, France) every two weeks from full bloom until maturity. The predawn Ψ_leaf_ was measured from eight leaves on eight individual vines at least 30 minutes before sunrise between 3:45 a.m. and 6:25 a.m. on each date. The predawn Ψ_leaf_ was assumed to be a proxy for soil water potential (Ψ_soil_) [73]. Midday Ψ_xylem_ was determined from eight fully expanded leaves on eight individual vines on midday between 2 p.m. and 4 p.m. and all leaves were covered with an opaque reflective plastic envelope for at least one hour before measurements to prevent transpiration and allow the Ψ_leaf_ to come into equilibrium with the Ψ_xylem_.

Berry FW and [Sugar] were collected weekly from veraison (i.e. the onset of ripening) to harvest each year from 2004 to 2016 (8–10 times for each parcel in each year), on a sample of 800 berries (eight berries per vine). Berry sugar content was determined with a handheld refractometer as described in van Leeuwen *et al.* [74]. Berry DW was estimated from a robust relationship between berry water content and [Sugar] that has been well established with various cultivars across multiple vintages [75]. Berry number per shoot was counted from 15 randomly selected primary shoots.

Canopy leaf area and shoot characteristics were measured in 2011. Firstly, the relationship between shoot leaf area and shoot length was established for primary and lateral shoots, respectively, based on measurements of 15 randomly selected shoots for each shoot type. Leaf area was measured using Li-Cor 3000 portable leaf area meter (LI-COR, Lincoln, NE, USA) and shoot length by tape. Then, 10 vines were randomly selected to measure the length and number of all primary and lateral shoots. Lastly, the mean number of primary shoots per vine, mean total leaf area per vine and mean length per primary shoot were calculated [72]. Additionally, 20 leaves of Cabernet Franc were sampled to determine the specific leaf area (SLA) by measuring the individual leaf area and dry weight, the leaf length/leaf width ratio and the relationship between leaf area and leaf length. The declination angle between the petiole and internode, and the declination angle between leaf blade and petiole were measured by protractor on 20 randomly selected single-shoot fruiting cuttings (10–13 leaves per shoot) of ‘Cabernet Sauvignon’ in 2015.

To avoid time trend effect during time-series analysis for the traits of xylem water potential, berry fresh weight, berry dry weight and berry sugar concentration, we used three methods to detrend the time series data. The ‘detrend’ function in R package ‘pracma’ was used to detrend data by fitting with a constant value and linear regression model, respectively. In addition, the data was detrended by differencing method using ‘diff’ function in R. The original data was then compared to the detrend data with linear fitting. Significant linear relationships (r^2^ closed to 1, *P* < 1e^−16^) were detected between original data and detrend data (results not shown), indicating that those original observed data are stationary and do not need to be detrended.

### Meta-analysis of leaf *P*_n_ and *g*_s_ responses to Ψ_leaf_ and midday Ψ_xylem_

Leaf *P*_n_ and *g*_s_ were not measured when measuring midday Ψ_xylem_ and predawn Ψ_leaf_ in the present study. To compare between simulated leaf gas exchanges and Ψ_leaf_, with observations and to derive stable model parameters under field conditions, simultaneously observed data of *P*_n_, *g*_s_, midday Ψ_leaf_, and/or predawn Ψ_leaf_ or Ψ_soil_ were collected from field grapevine studies published in peer-reviewed scientific journals and conference proceedings. Publications were reviewed and selected based on the following criteria: (i) only field studies were conserved when considering the potential utilization of the current model for field vineyard management and significant differences of *g*_s_ measured between field and greenhouse conditions [43]; and (ii) presence of both leaf *P*_n_ or *g*_s_ and predawn and midday Ψ_leaf_ or predawn Ψ_soil_ values matched in time either measured seasonally or diurnally. For data under graphical representation, a free tool ‘web plot digitizer’ (https://automeris.io/WebPlotDigitizer/) was used to retrieve values. In total, 18 studies were selected and used (Table S1 and Dataset S1, see online supplementary material).

### Model inputs and initial conditions

Climate data (total solar radiation, temperature, relative humidity, and wind speed) collected by a weather station located in the experimental vineyard were used as model input. Predawn Ψ_leaf_ was inputted as proxy for Ψ_soil_ and was bi-weekly collected in the current study (see section: Experimental data). Predawn Ψ_leaf_ between two field measurement dates was estimated using linear interpolation.

The initial state of the grapevine at veraison included canopy architecture, berry properties, and dry mass of all organ compartments. Following the evidence that there is vessel segmentation among shoots and water flows in discrete xylem sectors along the trunk axis [9, 62], the canopy architecture was represented by several isolated plants with a single shoot and without consideration of possible water fluxes between annual shoots. To mimic the actual canopy architecture, the shoot density and its geometric organization was set to the same as vines grown in the field with a pruning system of one-cane cordon. These geometric configurations included shoot number per meter, mean shoot length, mean leaf number per shoot, and leaf area per shoot, and they were all estimated based on measurements from experimental data. The distance between the two consecutive single-shoot plant was set to 15 cm. Leaf area along the shoot was multiplied by a factor to give the observed leaf area per shoot in field based on the node-by-node leaf area measurements in Cabernet Franc [76]. Leaf length was estimated based on the relationship between leaf area and leaf length and leaf width was estimated based on leaf length/width ratio (see section: Experimental data). The declination angle between the petiole and internode, and the declination angle between leaf blade and petiole were determined based on measurements from Cabernet Sauvignon (see section: Experimental data). The leaf azimuth angle between consecutive leaves in current simulations was set to 135°. Leaf dry mass was estimated by leaf area and SLA. Leaf area per shoot was set to 0.25 m^2^. Shoot and fine root dry mass were estimated according to the measurements of Hunter [77]. Wood dry mass was estimated by allometric relationships between vine age and trunk diameter [78], and between trunk diameter and biomass [79]. Structural root biomass was estimated based on Hunter [77] and added to the trunk biomass because structural roots perform similar carbohydrate cycles as the trunk [80]. The dry mass of trunk and fine root per vine were evenly distributed to each single-shoot plant based on the mean shoot number per vine. To initialize the berry growth, the day of the year (DOY) of veraison and harvest, berry [Sugar], FW, DW, and mean berry number per shoot at veraison were determined based on experimental data (see section: Experimental data). The external canopy architecture is fixed without new modifications from veraison to maturity. The initial state of canopy architecture and dry masses of organs (except berry) were assumed to be the same among the year, while berry traits at veraison were extracted from the experimental data at the beginning of the season for each year.

### Model parameterization

A comprehensive description of parameterization and validation for several grape cultivars of potted vines growing in controlled greenhouse conditions has been reported in Zhu *et al.* [22, 23]. In the present study, the model needs to be recalibrated for field-growing vines with distinct above-ground sizes and root systems compared to the potted vines. These differences can influence the values of 14 model key parameters, which have been shown to exert high impact on model outputs according to previous sensitivity analysis [23, 38]. These 14 parameters were newly recalibrated, mostly related to the resistance between the rhizosphere and soil–root interface (*R*_sp_), leaf photosynthesis activities, and berry growth, while the remaining 104 parameters were kept as those previously reported [23, 38]. The simulation started at veraison and ended at harvest determined by filed berry sampling (see section: Experimental data). The 14 recalibrated parameters were described in more detail as below.

### Soil-to-root water transport

Five parameters involved in the *R*_sp_ (mg MPa^−1^ s^−1^ plant^−1^). *R*_sp_ plays a key role in determining water transport from soil to root and is determined by soil hydraulic conductivity (*k*(Ψ_soil_)) and root system architecture and was calculated as in Gardner [81]:

(1) \begin{equation*} {R}_{sp}=\frac{\ln \left({d}^2/{r}^2\right)}{4\pi k\left({\psi}_{soil}\right){L}_a}=\frac{RSC}{k\left({\psi}_{soil}\right)} \end{equation*}

where the *d*, *r* and *L_a_* are the mean distance between neighboring roots, root radius and root length per unit of area, respectively. Considering the *d*, *r* and *L_a_* were not measured and are extremely hard to measure under field conditions, this part was simplified and represented by a novel parameter root specific conductance (*RSC*) in the current model (Eq. 1). The *RSC* was parameterized at the whole plant-level by maximizing the sum of log-likelihood of the simulated Ψ_xylem_ and the observed Ψ_xylem_ using the random walk Markov chain Monte Carlo (MCMC) method [82]. This method assumes the prior parameter values and the observations follow a Gaussian distribution and was carried on automatically with a self-written Java program in GroIMP.

The *k*(Ψ_soil_) represents soil hydraulic conductivity and is related to Ψ_soil_. The equation from van Genuchten [83] (1980) with four parameters was used as follows:

(2) \begin{align*} k\left({\psi}_{soil}\right)&= ks{\left(\frac{1}{1+{\left({\alpha}_v\times{\psi}_{soil}\right)}^n}\right)}^{\left(p-p/n\right)}\notag\\ & \quad\times{\left(1-{\left(1-\frac{1}{1+{\left({\alpha}_v\times{\psi}_{soil}\right)}^n}\right)}^{\left(1-1/n\right)}\right)}^2 \end{align*}

where *ks* (saturated soil hydraulic conductivity), *n*, *α_v,_* and *p* are coefficients that characterize a given soil type. The parameters in Eq. 2 were estimated with data from Duursma *et al.* [40] for silt clay loam soil.

### Leaf gas exchange

Three parameters, including *leafN_content*, *slope_V_cmax_*, and Ψ_50%-leaf_ involved in leaf gas exchange. The *leafN_content* exerts impact on the leaf maximum carboxylation rate and it was determined according to measurements in the field [10]. The *slope_V_cmax_* represents the slope of the linear regression between the maximum carboxylation rate and *leafN_content*, and its value was parameterized using the same method as that used to parameterize the *RSC.* The Ψ_50%-leaf_ is the leaf water potential when 50% of the leaf conductivity is lost and can influence leaf *g*_s_ prediction in the Tardieu–Davies module. As the value of Ψ_50%-leaf_ that measured in previous study resulted in a steep decline of *g*_s_ under increasing soil water stress, it was finely calibrated to match the observed data based on expert knowledge for field growing vines.

### Berry water and carbon balance

Six parameters related to berry water and carbon balances in the water transport and carbon allocation modules were parameterized through whole-plant model optimization with a calibration data set. Firstly, the maximal rate of active sugar uptake per unit of dry mass (*V_max, berry_*) and Michaelis constant for active transport of sugar per unit water (*K_m, berry_*), both controlling berry sugar active importation rate, were parameterized at the whole-plant level by maximizing the sum of log-likelihood of the simulated model outputs versus the observed berry DW using the random walk MCMC method. Then maximal phloem hydraulic conductance (*L_p, max_*), minimal phloem hydraulic conductance (*L_p, min_*), fresh mass at the inflection point (FM^*^_Lp,_) and proportional to the slope at inflection point of phloem hydraulic conductance (*k_Lp_*), which represent berry phloem water conductivity and control berry water uptake, were parameterized by optimizing the log-likelihood of the simulated model outputs versus the observed berry FW and [Sugar].

Parameterization was conducted with a calibration data set, which included experimental observations from vintages of 2005, 2009, 2011, and 2013, while the remaining data of nine vintages were used for the model validation (Table S2, see online supplementary material). The four calibration vintages were chosen based on their climate and soil water status to represent all 13 vintages. As a result, the ranges of climate and Ψ_soil_ were similar between calibration and validation data sets (Table S2, see online supplementary material). The parameter optimization for the whole plant water flux and berry growth were done at the computing facilities MCIA (Mésocentre de Calcul Intensif Aquitain, on the cluster Curta) of the University of Bordeaux due to the large computation power needed. All parameters that were reparametrized in this work were listed (Table S3, see online supplementary material).

The model performance for the calibration data set in simulating Ψ_xylem_, berry DW, FW, and [Sugar] was evaluated in terms of the following statistical indices: root mean square error (RMSE, Eq. 3), and the relative root mean square error (RRMSE, Eq. 4), indicators of the overall relative accuracy of a model.

(3) \begin{equation*} RMSE=\sqrt{\frac{\sum \limits_{i=1}^n{\left({O}_i-{S}_{\mathrm{i}}\right)}^2}{\mathrm{n}}} \end{equation*}

%

(4) \begin{equation*} RRMSE=\frac{RMSE}{\overline{O}}\times 100 \end{equation*}

where *O_i_* is the observed values and *S_i_* is the simulated values. The smaller the RMSE and RRMSE, the more accurate the simulation [84]. In this study, model accuracy is considered excellent when RRMSE <10%; good if 10% ≤ RRMSE <20%; fair if 20% ≤ RRMSE <30%; and poor if RRMSE ≥30% [85].

### Model validation

After parameterization, the model was validated using the data set of nine vintages independent of calibration. Simulations were run with berry FW, DW, and [Sugar] at beginning of the veraison, phenology date of veraison and ripening, and meteorological data and predawn Ψ_leaf_ along the season after veraison for each year as inputs. The performance of the model in simulating leaf *g*_s_ and *P*_n_ was assessed by comparing simulated responses of *g_s_* and *P*_n_ to predawn or midday Ψ_leaf_ to the same response curves drawn from the meta-analysis. The performance of the model in simulating Ψ_xylem_, berry DW, FW, and [Sugar] was evaluated by comparing model outputs with experimental observations with RMSE and RRMSE.

### Scenario simulations

The advanced phenology due to climate change will result in berry ripening during warmer periods [30, 31, 34, 54, 55]. A virtual scenario analysis was conducted and analysed to test the effects of advanced phenology on fruit development. The simulations starting from observed date of veraison to observed date of harvest of each vintage were regarded as the default scenario. Considering the observed advancement of veraison in the past decades [30, 31, 34, 54, 55], two virtual simulation scenarios were investigated, including a 14-day (scenario eVer_14) or 28-day (scenario eVer_28) advancement of veraison and maturity earlier compared to the default scenario examined in this work. The climate and predawn Ψ_leaf_ inputs were extracted from the same file as those for the default veraison scenario, but the inputted time window was shifted to synchronize with the virtual veraison scenarios, by maintaining the duration of simulation. Except for the date of veraison and end of simulation, the other initial settings were kept the same as in the default veraison scenario. All scenarios were simulated for 13 vintages.

### Statistical analyses

The effect of early veraison on berry FW, DW, and [Sugar] was analysed with one-way ANOVA. To test if the slopes and intercepts of linear regression relationships between observed and simulated values were different from 1:1 line, the ANCOVA test was performed. The contribution of predawn Ψ_leaf_ and climate factors (radiation, daily maximum temperature, VPD) to Ψ_xylem_ was calculated using the ‘*calc.relimp*’ function of the ‘*relaimpo*’ R package. Statistical analyses were performed using R software [86].

## Acknowledgments

The authors thank Nabil Girollet (INRAE, Bordeaux, France) and Pierre Gay (Université de Bordeaux) for assistance in setting and running the model on the computing facilities MCIA (Mésocentre de Calcul Intensif Aquitain, on the cluster of Curta) of the University of Bordeaux. W.Y. thanks the Chinese Scholarship Council (CSC) for supporting his study in INRAE, France. We are grateful to multiple interns who assisted with data collection.

This research was supported partly by National Key R&D Program of China (grant numbers 2021YFE0109500, 2019YFD1000100), CAS Youth Interdisciplinary Team (JCTD-2022-06), and National Natural Science Foundation of China (grant number 31860527). Research was conducted as part of the LIA INNOGRAPE International Associated Laboratory.

## Author contributions

W.Y.: data curation, formal analysis, investigation, visualization, methodology, writing-original draft; J.Z.: methodology, software, writing-review and editing; C.vL.: data collection, resources, investigation, writing-review and editing; Z.D.: conceptualization, funding acquisition, supervision, writing-review and editing; G.A.G.: conceptualization, supervision, writing-review and editing.

## Data availability

All data supporting the findings of this study are available within the paper and within its supplementary materials published online.

## Conflict of interest statement

None declared.

## Supplementary data

Supplementary data is available at *Horticulture Research* online.

## Supplementary Material

Web_Material_uhad071
