# Supplementary material for: GrapevineXL reliably predicts multi-annual dynamics of vine water status, berry growth, and sugar accumulation in vineyards
Source: Hortic Res. 2023 Apr 13;10(6):uhad071. doi: 10.1093/hr/uhad071 (PMC10244804; doi:10.1093/hr/uhad071)
Supplement: Web_Material_uhad071 [file web_material_uhad071.zip › Revised_supplemtaryFiles_vfin_HR_3.29.docx]

**Supplementary files**

**Table S1** List of references used to extract and build the dataset for calibrating the model gas exchange module. A series of relationships were established, including these between predawn leaf water potential (Ψ_leaf_) and stomatal conductance (*g*_s_), between predawn Ψ_leaf_ and net photosynthesis rate (*P*_n_), between midday Ψ_leaf_ and *g*_s_ and between midday Ψ_leaf_ and *P*_n_, respectively.

| **Order** | **References** | **Source** | **Scions** | **Rootstocks** | **Method** | **Water potential type** |
| --- | --- | --- | --- | --- | --- | --- |
| 1 | Alsina, M.M. *et al.* 2011 | Journal of Experimental Botany, 2011, Vol. 62, No. 1, pp. 99–109 | Merlot | 101-14 Mgt, 1103P | IRGA | Midday leaf water potential |
| 2 | Centeno, A. *et al.* 2010 | HortTechnology, 2010, Vol. 20, No. 3, pp. 585–593 | Tempranillo | 110R | IRGA | Midday leaf water potential |
| 3 | Schultz, H.R. 1996 | Acta Horticulturae, 1996, Vol. 427, pp. 251–256 | Syrah, Grenache | 110 Richter, 140 Ruggeri | IRGA | Midday leaf water potential |
| 4 | de Souza, C.R. *et al.* 2005 | Agriculture, Ecosystems and Environment, 2005, Vol. 106, pp. 261–274 | Castelão, Muscat of Alexandria | 1103P | IRGA | Predawn and midday leaf water potential |
| 5 | Flexas, J. *et al.* 1998 | Australian Journal of Plant Physiology 1998, Vol. 25, 893–900 | Tempranillo | 110R | IRGA | Predawn and midday leaf water potential |
| 6 | Giorio, P. and Nuzzo, V. 2012 | Plant Biosystems, 2012, Vol. 146, No. 2, pp. 322–333 | Montepulciano | 1103P | IRGA | Midday leaf water potential |
| 7 | Intrigliolo, D.S. and Castel, J.R. 2009 | Agricultural water management, 2009, Vol. 96, 282–292 | Tempranillo | 161-49C | Porometer | Predawn and midday leaf water potential |
| 8 | Loveys, B.R. and Düring, H. 1984 | New Phytologist, 1984, Vol. 97, No. 1, pp. 37–47 | Riesling, Sylvaner | Own-rooted | Porometer | Midday leaf water potential |
| 9 | Medrano, H. *et al.* 2003 | Functional Plant Biology, 2003, Vol. 30, No. 6, pp. 607–619 | Tempranillo, Manto negro | 110R | IRGA | Predawn leaf water potential |
| 10 | Moutinho-Pereira, J.M. *et al.* 2004 | Photosynthetica, 2004, Vol. 42, No. 1, pp. 81–86 | Touriga nacional | 1103P | IRGA | Midday leaf water potential |
| 11 | Rodrigues, M.L. *et al.* 2008 | Functional Plant Biology, 2008, Vol. 35, pp. 565–579 | Castelão, Muscat of Alexandria | 1103P | Porometer | Predawn leaf water potential |
| 12 | Speirs, J. *et al.* 2013 | Journal of Experimental Botany, 2013, Vol. 64, No.7, pp. 1907–1916 | Cabernet Sauvignon | Ramsey | Porometer | Midday leaf water potential |
| 13 | Baeza, P. *et al.* 2007 | Scientia Horticulturae, 2007, Vol.114, No.3, pp. 151-158 | Cabernet-Sauvignon | SO4 | IRGA | Midday leaf water potential |
| 14 | Prieto, J.A. *et al.* 2010 | OENO One, 2010, Vol. 44, No.1, pp. 9–20 | Syrah, Marselan, Grenache, Mouredre, Ekigaina | 140Ru | IRGA | Predawn leaf water potential |
| 15 | Levin, A.D. *et al.* 2019 | Functional Plant Biology, 2019, Vol.47, No.1, pp. 11–25 | Average over 17 cultivars | 1103P | Porometer | Midday leaf water potential |
| 16 | Schultz, H.R. 2003 | Plant Cell & Enviorment, 2003, Vol.26, No.8, pp.1393–1405 | Syrah, Grenache | Rupestris*Berlandieri | IRGA | Predawn and midday leaf water potential |
| 17 | Zufferey, V. and Smart. D. 2012 | Functional Plant Biology, 2012, Vol.39, No.12, pp. 1019–1027 | Syrah | Vitis riparia *Vitis rupestris | IRGA | Predawn and midday leaf water potential |
| 18 | Beis, A. and Patakas, A. 2010 | Functional Plant Biology, 2010, Vol.37, No.2, pp. 139–146 | Sabatiano and Mavrodafni | Own-rooted | IRGA | Predawn leaf water potential |

**Table S2** Summary information for climate and soil water potential between veraison and harvest for all observed years in the vineyard in Saint-Emillion, Bordeaux.

| Year | Type of data set | Number of days with Tmax > 30 °C | Number of days with Tmax > 25 °C | Total Solar Radiation (× 10^4^ J m^-2^) | Total grow degree days (°C days) | Precipitation (mm) | Number of days with rain | Mean temperature (°C) | Minimum predawn leaf water potential (MPa) | Maximum predawn leaf water potential (MPa) |
| --- | --- | --- | --- | --- | --- | --- | --- | --- | --- | --- |
| 2004 | Validation | 5 | 23 | 7.48 | 435 | 61 | 13 | 19.7 | -0.56 | -0.17 |
| 2005 | Calibration | 12 | 34 | 8.69 | 474 | 63 | 12 | 20.3 | -0.72 | -0.39 |
| 2006 | Validation | 10 | 28 | 7.83 | 477 | 160 | 24 | 20.4 | -0.72 | -0.38 |
| 2007 | Validation | 8 | 25 | 8.75 | 434 | 90.4 | 27 | 19.1 | -0.30 | -0.11 |
| 2008 | Validation | 2 | 15 | 8.47 | 389 | 108 | 39 | 17.2 | -0.29 | -0.17 |
| 2009 | Calibration | 11 | 36 | 9.15 | 541 | 107 | 25 | 20.2 | -0.55 | -0.18 |
| 2010 | Validation | 7 | 32 | 9.16 | 463 | 52 | 20 | 19.1 | -0.47 | -0.03 |
| 2011 | Calibration | 12 | 35 | 8.93 | 575 | 103 | 31 | 21.2 | -0.37 | -0.03 |
| 2012 | Validation | 15 | 33 | 8.95 | 536 | 52.4 | 17 | 20.1 | -0.45 | -0.20 |
| 2013 | Calibration | 6 | 21 | 6.97 | 444 | 129 | 32 | 19.1 | -0.30 | -0.11 |
| 2014 | Validation | 3 | 31 | 9.55 | 518 | 133 | 33 | 19.6 | -0.28 | -0.17 |
| 2015 | Validation | 11 | 25 | 9.55 | 528 | 105 | 37 | 19.4 | -1.06 | -0.10 |
| 2016 | Validation | 20 | 40 | 9.27 | 581 | 69.1 | 16 | 21.0 | -0.67 | -0.01 |
| Coefficient of variance (CV, %) | | 53.02 | 24.09 | 9.18 | 12.03 | 35.70 | 35.98 | 5.25 | 44.14 | 74.62 |

**Table S3** Values, units, and sources of inputs for the initial state of canopy architecture and the 14 parameters that newly parametrized and used in the study.

| Symbol | Description | Values used in the study | Sources |
| --- | --- | --- | --- |
| Inputs for the initial state of canopy architecture | | | |
| BIOMASS_INTERNODE | internode biomass | 20 g | Hunter 1998 |
| BIOMASS_WOOD | Wood and structural root biomass | 310 g | Miranda et al. (2017), Nader (2019) and Hunter 1998 |
| BIOMASS_ROOT | fine root biomass | 10 g | Hunter 1998 |
| SLA | specific leaf area | 190 cm^2^ g^-1^ | Experiment |
| *Shoot_length* | Mean one-year-old shoot length | 1 m | Experiment |
| *plantDistance* | Distance between plants in a row | 0.15 m | Experiment |
| *WOOD_LENGTH* | Length of the perinial wood part | 70 cm | Experiment |
| *MAX_PHYTOMER_NUM* | total phytomer number | 23 | Experiment |
| *MAX_LEAF_NUMBER* | Total leaf number | 20 | Experiment |
| *STARTING_LEAF_NUMBER* | Phytomer rank where the first leaf positioned | 3 | Experiment |
| Leaf size | leaf length and width distribution along shoot | - | Sanchez-de-Miguel et al. (2011) |
| Soil hydrodynamic properties | | | |
| *n* | coefficients that characterize a given soil | 1.08 (Dimensionless) | Duursma et al. 2007 |
| *α_v_* | coefficients that characterize a given soil | 8.13 (Dimensionless) | Duursma et al 2007 |
| *Ks* | soil hydraulic conductivity at saturation | 1.34 Kg m^-1^ s^-1^ Pa^-1^ | Duursma et al 2007 |
| *p* | coefficients that characterize a given soil | 1.26 (Dimensionless) | Duursma et al 2007 |
| *RSC* | specific root conductance | 6.06e-6 | calibration |
| Phloem hydraulic conductance | | | |
| *L_p,max_* | minimal phloem hydraulic conductance | 1.54e-2 g cm^-2^ MPa h^-1^ | calibration |
| *L_p_,_min_* | maximal phloem hydraulic conductance | 6.39e-06 g cm^-2^ MPa h^-1^ | calibration |
| *K_Lp_* | Fresh mass at the inflection point | 6.72 g^-1^ | calibration |
| *FM*_Lp_* | proportional to the slope at the inflection | 8.68e-1 g | calibration |
| Sugar uptake-active uptake | | | |
| *V_max,berry_* | maximum uptake rate of carbon per unit of dry mass | 1.63e-2 g (gDW)^-1^ h^-1^ | calibration |
| *K_M,berry_* | Active uptake of dry material (Michaelis Menten) | 0.04 g (gH_2_O)^-1^ | calibration |
| Leaf hydraulic conductance traits | | | |
| *Ψ_50%_* | leaf water potential when 50% of the leaf conductivity is lost | -1.8 MPa | calibration |
| Leaf photosynthesis | | | |
| *V_cmax_* | slope for the relationship between maximum carboxylation rate and leaf nitrogen | 49.54 | calibration |
| *leafN_conten*t | leaf nitrogen content per leaf area | 2.1 g m^-2^ | Prieto et al. (2012) |

**
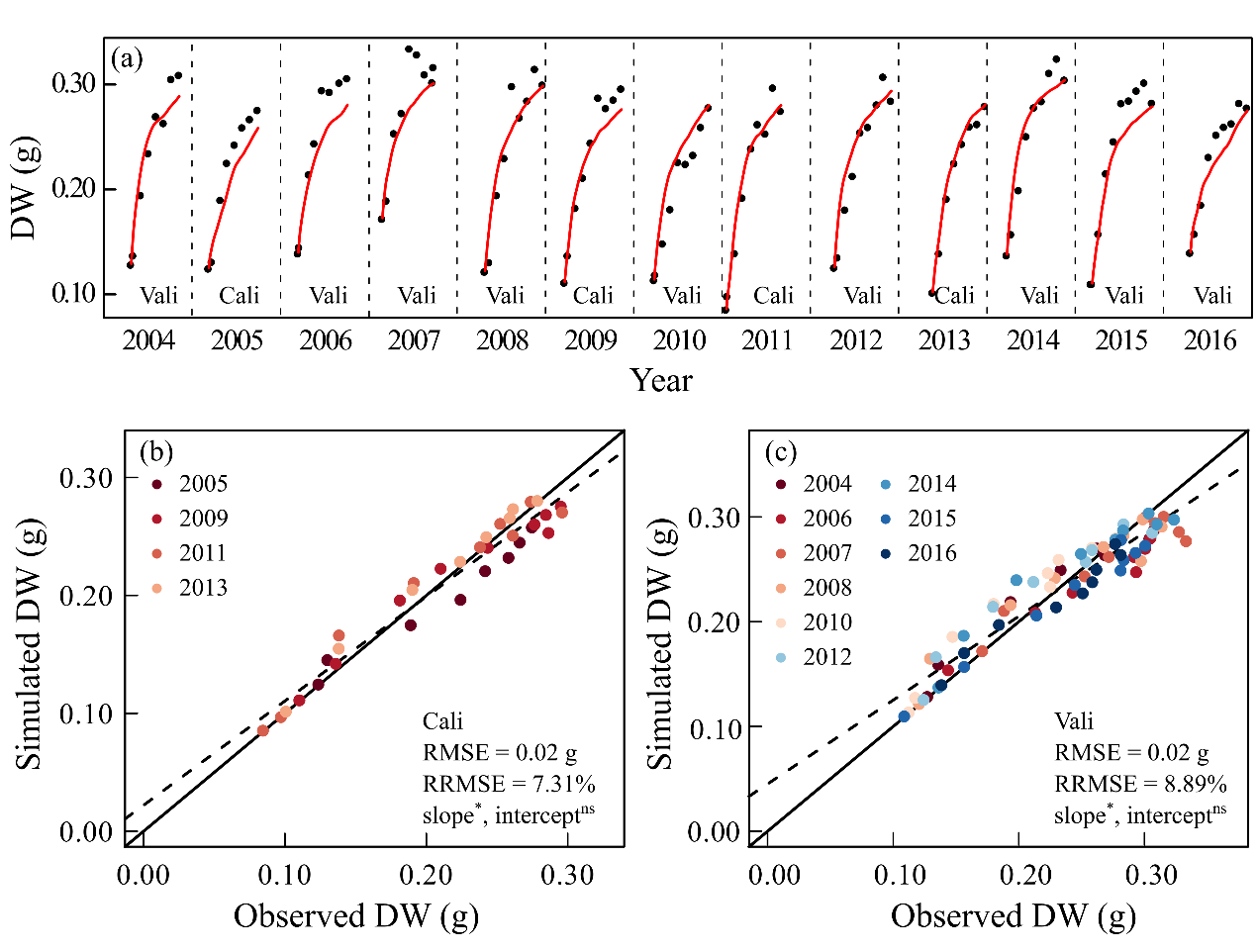
**

**Fig. S1** Observed and simulated berry dry weight (DW) in field from 2004 to 2016. Cali and Vali represent the data belonging to calibration (2005, 2009, 2011, and 2013) and validation (the remaining 9 years) data sets, respectively. The upper panel showed comparisons between observed and simulated berry DW over time, and below panels were direct comparisons for calibration (b) and validation (c) years, respectively. Each point represented the average value of all berries from the simulated vine. Black points in upper panels are observed mean berry DW and solid lines are simulated values. Solid lines in bottom panels are the 1:1 lines between observed and simulated values, and dashed lines are the linear regression lines. * and ns represent significant or no significant difference in slope and intercept detected between regression line of observed and simulated values and 1:1 line at p < 0.05, using ANCOVA.


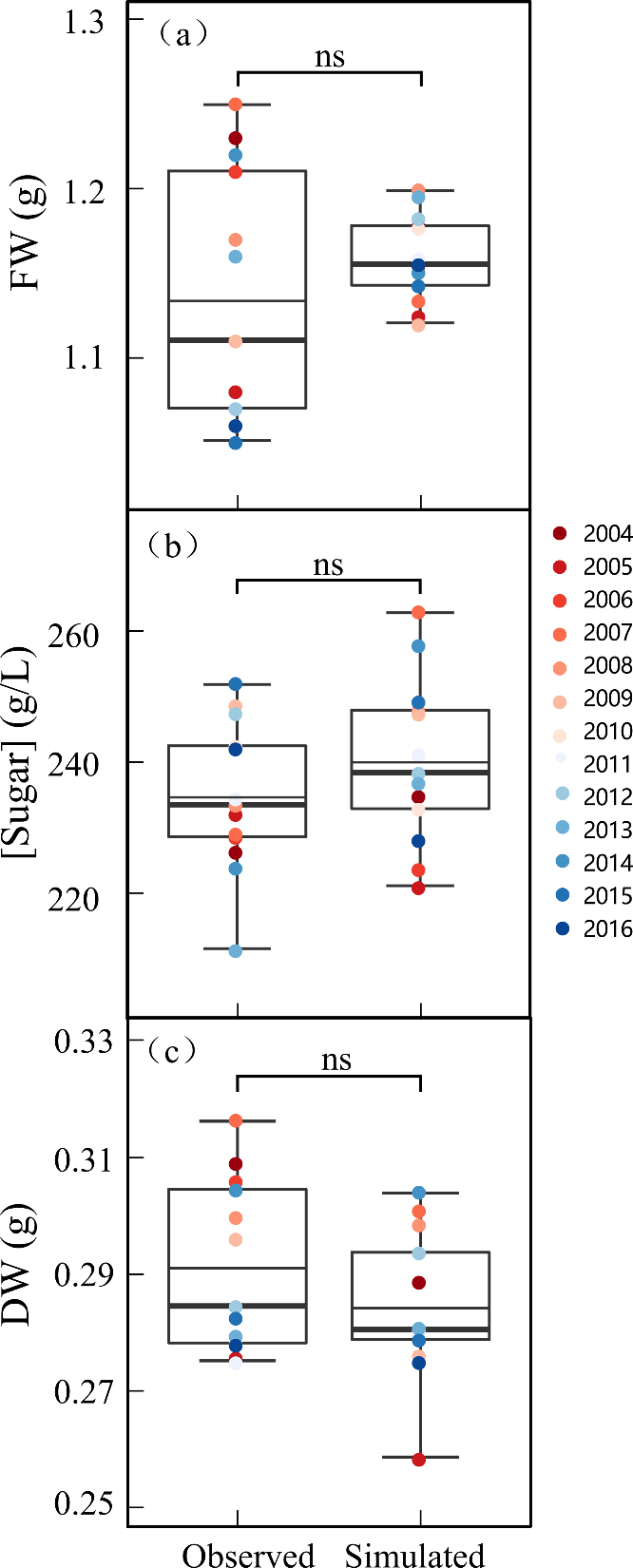


**Fig. S2** Observed and simulated berry fresh weight (FW), sugar concentration ([Sugar]) and dry weight (DW) at harvest over 13 years from 2004 to 2016, using GrapevineXL model in field. The boxes represent the 25-to-75 percentile, whiskers (i.e., the horizontal dashes linked to boxes by vertical lines) represent the 10-to-90 percentile, thick and thin dashes in boxes are median and mean of all values, and colorful dots are observed or simulated results. ns represents no significant differences were detected between observed and simulated values at *p* < 0.05, using one-way ANOVA.


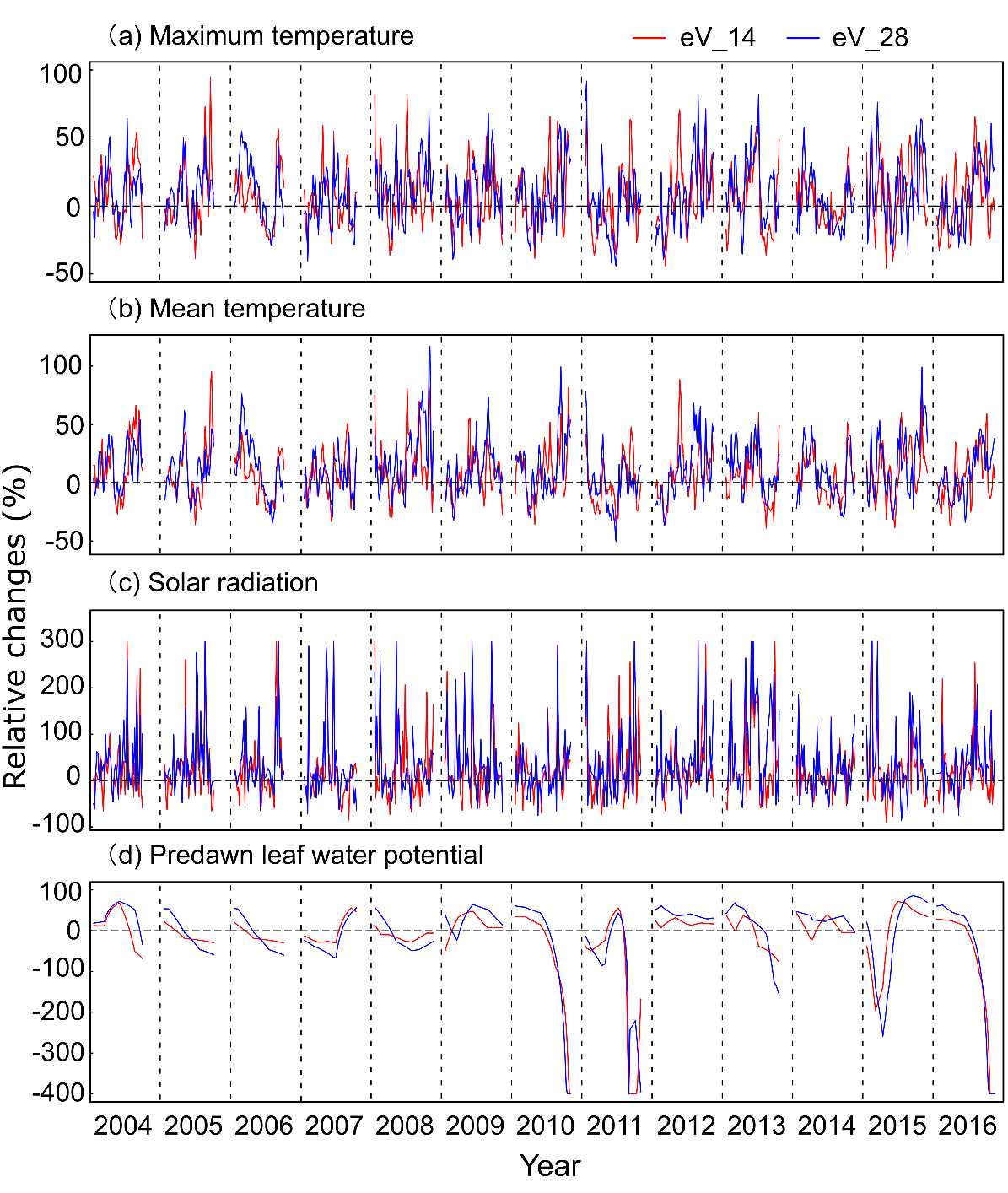


**Fig. S3** Relative changes in maximum temperature (a), mean temperature(b), solar radiation (c) and predawn leaf water potential (d) over 13 years from 2004 to 2016 under earlier veraison scenarios. The climate and soil water potential files under the default veraison condition were used as baseline to estimate the relative changes under 14- and 28-days earlier veraison scenarios, respectively. Default scenario represented scenario that model run with actual date of veraison and harvest. The eVer_14 and eVer_28 represent the model run with date of veraison and harvest earlier than default by 14- and 28-days.The red and blue lines represent 14- and 28-day earlier veraison scenarios, respectively.


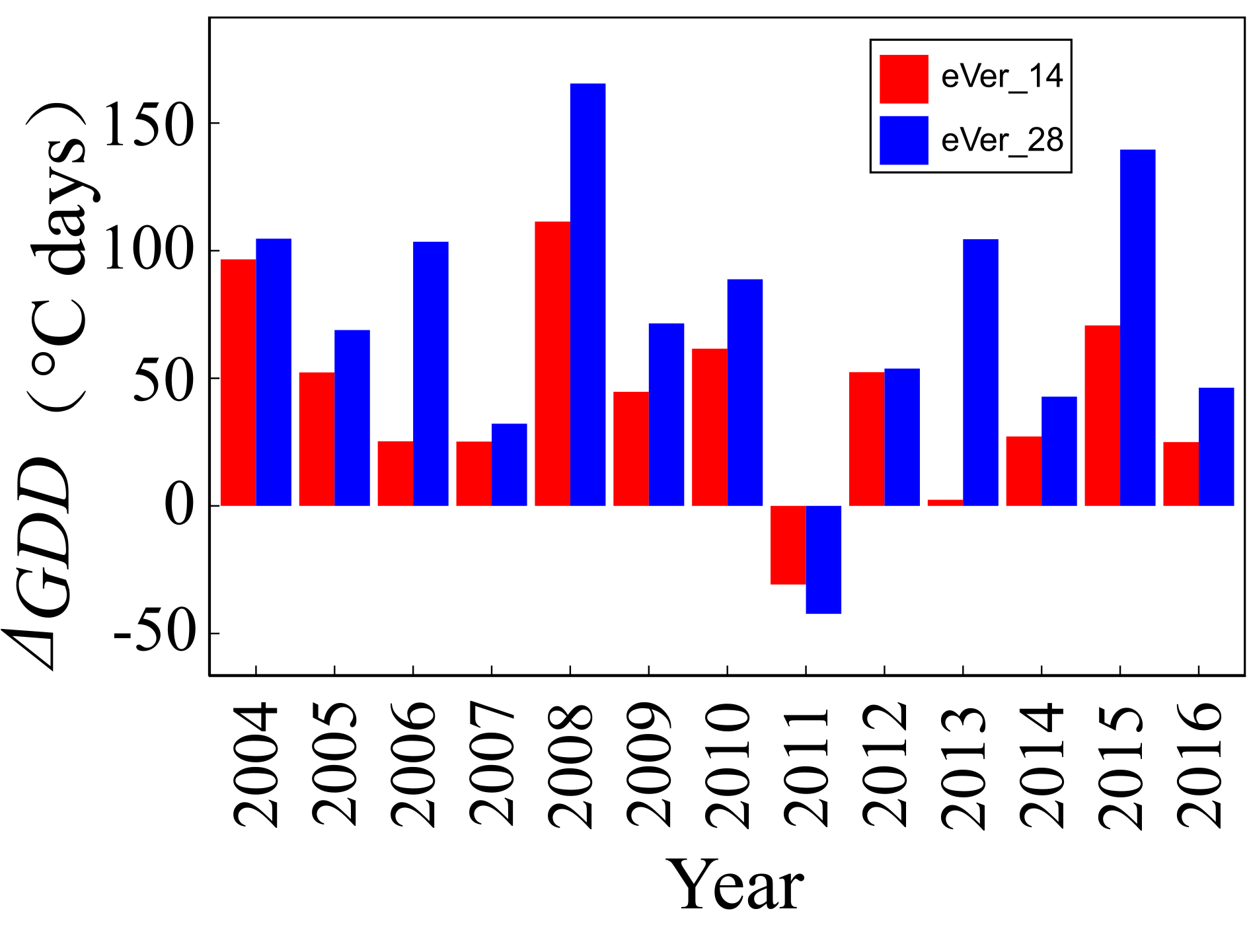


**Fig. S4** Relative changes of total growing degree day (*ΔGDD*) from veraison to end of simulation under earlier veraison scenarios compared to default veraison scenario over 13 years from 2004 to 2016. Default scenario represented scenario that model run with actual date of veraison and harvest. The eVer_14 and eVer_28 represent the model run with date of veraison and harvest earlier than default by 14- and 28-days. Red and blue bars represent the changes of GDD under 14- and 28-days earlier veraison scenarios, respectively.


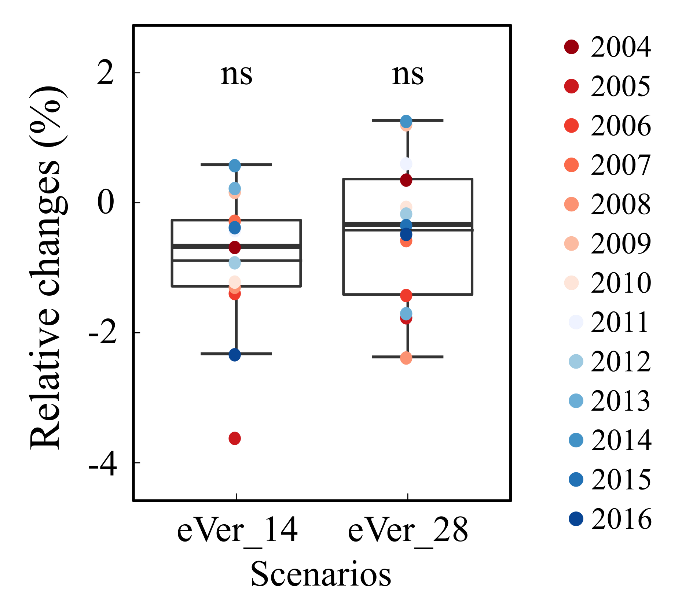


**Fig. S5** Relative changes in simulated berry dry weight over 13 years from 2004 to 2016, using GrapevineXL model in field, under earlier veraison scenarios. The simulated berry dry weight under default veraison were used as baseline in each year to estimate the relative changes corresponding to values simulated under 14- and 28-days earlier veraison scenarios, respectively. Default scenario represented scenario that model run with actual date of veraison and harvest. The eVer_14 and eVer_28 represent the model run with date of veraison and harvest earlier than default by 14- and 28-days. The boxes and whiskers represent the variation in relative changes among years. The thick and thin dashes in boxes are median and mean of all values, and colorful dots are simulated results. The ns represent no significant differences were detected between simulated values under default and earlier veraison scenarios at *p* < 0.05, using one-way ANOVA.


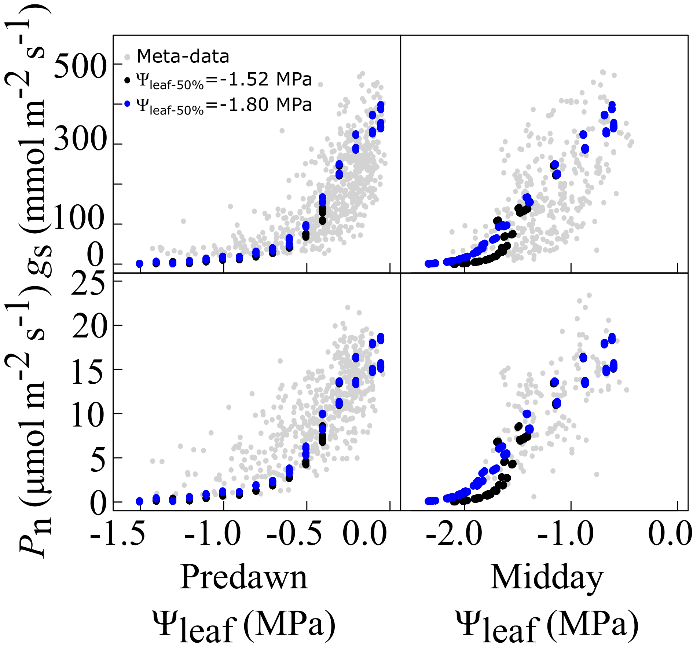


**Fig. S6** Observed and simulated responses of stomatal conductance (*g*_s_), and net photosynthesis rate (*P*_n_) to soil water potential (Ψ_soil_) and leaf water potential (Ψ_leaf_) using GrapevineXL model with different Ψ_leaf-50%_ (leaf water potential when 50% of the leaf conductivity is lost) settings. Grey points are a meta-dataset of published literature (Table S1), representing observed values. Black points are simulated values with Ψ_leaf-50%_ at -1.52 MPa while blue points with Ψ_leaf-50%_ at -1.80 MPa, respectively. All simulated values were extracted from the top three leaves of grapevine and model was ran under conditions with constant temperature (25 °C), photosynthetic active radiation (1 250 μmol m^-2^ s^-1^), carbon dioxide concentration (400 μmol/mol) and wind speed (1 m/s).


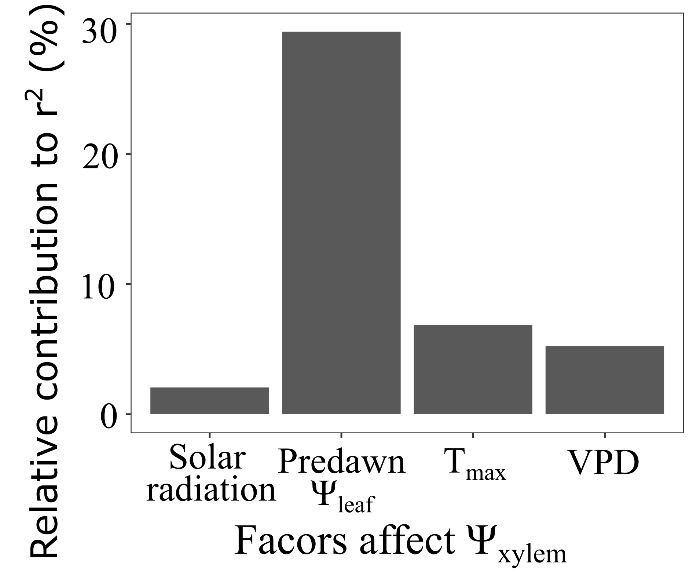


**Fig. S7** The contribution of solar radiation, predawn leaf water potential (Ψ_leaf_), maximum daily temperature (T_max_) and mean vapor pressure deficit (VPD) to xylem water potential (Ψ_xylem_). Contributions are expressed relatively to total model variance (r^2^).


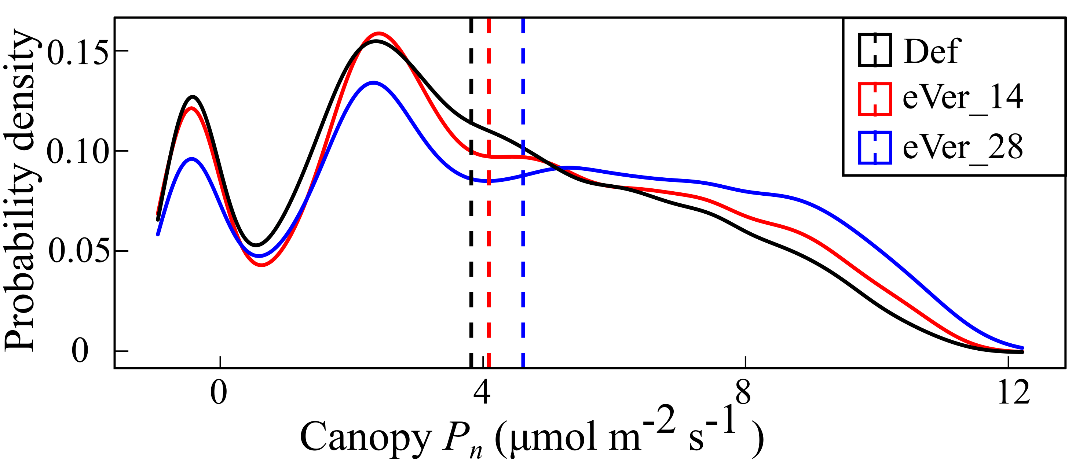


**Fig. S8** Simulated distribution of hourly canopy net photosynthesis rate (*P*_n_) within canopy over 13-years from 2004 to 2016 using GrapevineXL model in field, under default (black solid lines) and 14- (red solid lines) and 28-day (blue solid lines) earlier veraison scenarios, respectively. Def represents the default scenario that model run with actual date of veraison and harvest. The eVer_14 and eVer_28 represent the model run with date of veraison and harvest earlier than default by 14- and 28-days. Each curve corresponds to the analysis of a population of 316320 simulated values, which compose of hourly single leaf Pn of 20 leaves per vine at 24 hours per day, and various days over the post-veraison period, during the 13 years. Five vines were simulated with the configuration of field vine density, and only the vine located in the central part of the ‘field’ was used for the current analysis. The vertical lines present average canopy *P*_n_ over 13-years under default (black dashed lines), 14- (red dashed line) and 28-day (blue dashed line) earlier veraison scenarios, respectively.


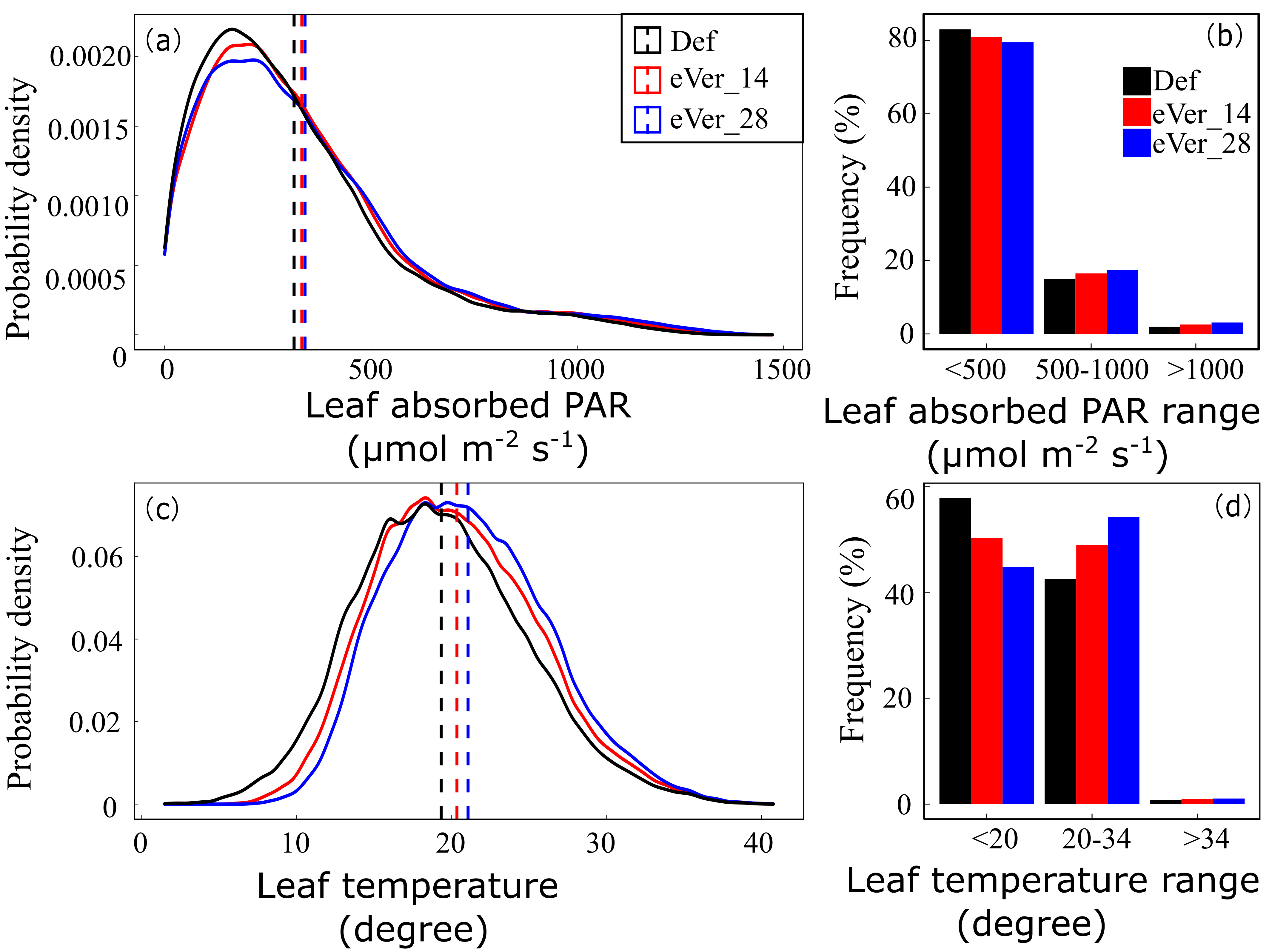


**Fig. S9** Simulated distribution of all single leaf absorbed photosynthetically active radiation (PAR, a) and leaf temperature (c) and frequencies of leaves corresponding to various range of leaf absorbed PAR (b) and temperature (d) within canopy over 13-years from 2004 to 2016 using GrapevineXL model in field, under default (black solid lines) and 14- (red solid lines) and 28-day (blue solid lines) earlier veraison scenarios, respectively. Def represents the default scenario that model run with actual date of veraison and harvest. The eVer_14 and eVer_28 represent the model run with date of veraison and harvest earlier than default by 14- and 28-days. The vertical lines present average leaf absorbed photosynthetically active radiation (a) and leaf temperature (b) over 13-years under default (black dashed lines), 14- (red dashed line) and 28-day (blue dashed line) earlier veraison scenarios, respectively


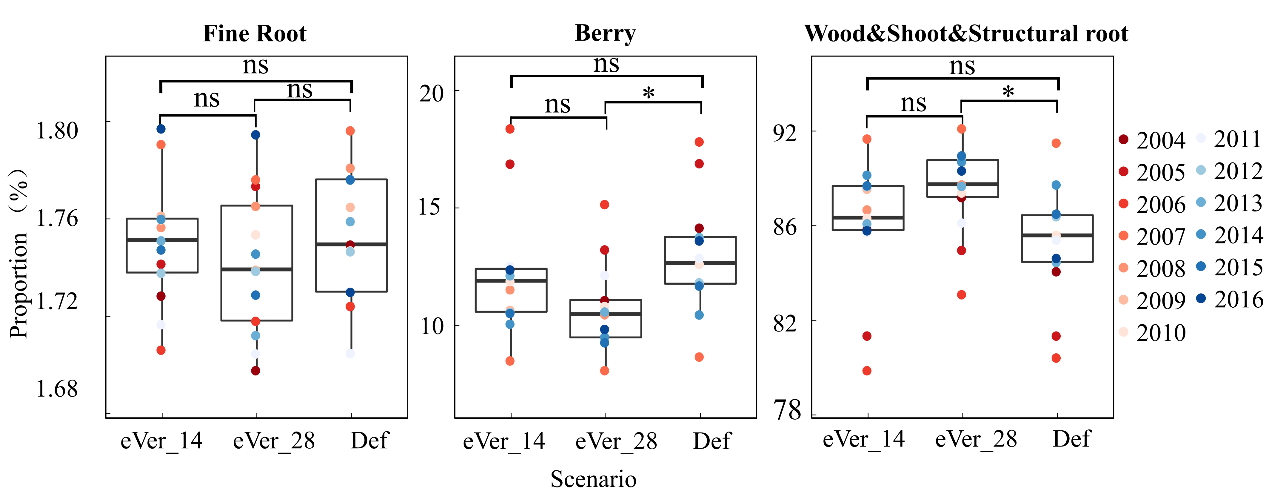


**Fig. S10** The proportion of carbon allocated among fine root, berry and structural part of vine including wood, shoot and structural root. Def represents the default scenario that model run with actual date of veraison and harvest. The eVer_14 and eVer_28 represent the model run with date of veraison and harvest earlier than default by 14- and 28-days. The ns represent no significant differences and * represent significant difference were detected between two compared scenarios at *p* < 0.05, using one-way ANOVA.
